# MTHFR Knockdown Assists Cell Defense against Folate Depletion Induced Chromosome Segregation and Uracil Misincorporation in DNA

**DOI:** 10.3390/ijms22179392

**Published:** 2021-08-30

**Authors:** Ming-Tsung Wu, Wei-Ting Ye, Yi-Cheng Wang, Po-Ming Chen, Jun-You Liu, Chien-Kuo Tai, Feng-Yao Tang, Jian-Rong Li, Chun-Chi Liu, En-Pei Isabel Chiang

**Affiliations:** 1 Department of Food Science and Biotechnology, National Chung Hsing University, Taichung 40227, Taiwan; mtlouiswu@gmail.com (M.-T.W.); chetbaker04567321@gmail.com (W.-T.Y.); Oraora169@yahoo.com.tw (Y.-C.W.); yaoming9@yahoo.com.tw (P.-M.C.); j31414jimmy5096@gmail.com (J.-Y.L.); 2 Department of Civil and Environmental Engineering, South Kensington Campus, Imperial College London, London SW7 2AZ, UK; 3 Department of Biomedical Sciences, National Chung Cheng University, Chia-Yi 62102, Taiwan; biockt@ccu.edu.tw; 4 Department of Nutrition, China Medical University, Taichung 40402, Taiwan; vincenttang@mail.cmu.edu.tw; 5 Institute of Genomics and Bioinformatics, National Chung Hsing University, Taichung 40227, Taiwan; fanicesiza@gmail.com (J.-R.L.); chunchiliu@gmail.com (C.-C.L.); 6 Innovation and Development Center of Sustainable Agriculture (IDCSA), National Chung Hsing University, Taichung 40227, Taiwan

**Keywords:** folate depletion, MTHFR, hepatocellular carcinoma, nucleotide biosynthesis, uracil misincorporation, micronuclei

## Abstract

Folate depletion causes chromosomal instability by increasing DNA strand breakage, uracil misincorporation, and defective repair. Folate mediated one-carbon metabolism has been suggested to play a key role in the carcinogenesis and progression of hepatocellular carcinoma (HCC) through influencing DNA integrity. Methylenetetrahydrofolate reductase (MTHFR) is the enzyme catalyzing the irreversible conversion of 5,10-methylenetetrahydrofolate to 5-methyltetrahydrofolate that can control folate cofactor distributions and modulate the partitioning of intracellular one-carbon moieties. The association between MTHFR polymorphisms and HCC risk is inconsistent and remains controversial in populational studies. We aimed to establish an in vitro cell model of liver origin to elucidate the interactions between MTHFR function, folate status, and chromosome stability. In the present study, we (1) examined MTHFR expression in HCC patients; (2) established cell models of liver origin with stabilized inhibition of MTHFR using small hairpin RNA delivered by a lentiviral vector, and (3) investigated the impacts of reduced MTHFR and folate status on cell cycle, methyl group homeostasis, nucleotide biosynthesis, and DNA stability, all of which are pathways involved in DNA integrity and repair and are critical in human tumorigenesis. By analyzing the TCGA/GTEx datasets available within GEPIA2, we discovered that HCC cancer patients with higher MTHFR had a worse survival rate. The shRNA of *MTHFR* (shMTHFR) resulted in decreased MTHFR gene expression, MTHFR protein, and enzymatic activity in human hepatoma cell HepG2. shMTHFR tended to decrease intracellular *S*-adenosylmethionine (SAM) contents but folate depletion similarly decreased SAM in wildtype (WT), negative control (Neg), and shMTHFR cells, indicating that in cells of liver origin, shMTHFR does not exacerbate the methyl group supply in folate depletion. shMTHFR caused cell accumulations in the G2/M, and cell population in the G2/M was inversely correlated with MTHFR gene level (r = −0.81, *p* < 0.0001), MTHFR protein expression (r = −0.8; *p* = 0.01), and MTHFR enzyme activity (r = −0.842; *p* = 0.005). Folate depletion resulted in G2/M cell cycle arrest in WT and Neg but not in shMTHFR cells, indicating that shMTHFR does not exacerbate folate depletion-induced G2/M cell cycle arrest. In addition, shMTHFR promoted the expression and translocation of nuclei thymidine synthetic enzyme complex SHMT1/DHFR/TYMS and assisted folate-dependent de novo nucleotide biosynthesis under folate restriction. Finally, shMTHFR promoted nuclear MLH1/p53 expression under folate deficiency and further reduced micronuclei formation and DNA uracil misincorporation under folate deficiency. In conclusion, shMTHFR in HepG2 induces cell cycle arrest in G2/M that may promote nucleotide supply and assist cell defense against folate depletion-induced chromosome segregation and uracil misincorporation in the DNA. This study provided insight into the significant impact of MTHFR function on chromosome stability of hepatic tissues. Data from the present study may shed light on the potential regulatory mechanism by which MTHFR modulates the risk for hepatic malignancies.

## 1. Introduction

The genotoxic consequences of folate insufficiency [1], either through reduced folate consumption or functional folate deficiency due to genetic defects in folate-related genes, contribute to numerous pathological conditions in humans, including cancer [2,3]. Folate mediated one-carbon metabolism has been suggested to play a key role in carcinogenesis and progression of hepatocellular carcinoma (HCC) through influencing DNA integrity [2,4,5].

### 1.1. Folate Depletion Causes Chromosome Instability

Deoxynucleoside triphosphates (dNTPs) are essential for the replication and maintenance of genomic stability. Regulation of cellular thymidylate synthesis is essential for DNA replication and genome stability in the nucleus [6]. Impaired de novo thymidylate synthesis due to folate deficiency results in deoxyuridine/uracil misincorporation into DNA and causes genome instability [7,8]. Folate depletion causes chromosomal instability by increasing DNA strand breakage, uracil misincorporation, and defective repair. Human lymphocytes cultured in folate-deficient media exhibit DNA double strand breaks [9], reduced DNA repair [9], and micronuclei formation [10,11,12]. High dietary folate consumption is associated with a lower micronucleus frequency in humans [13].

### 1.2. Folate Metabolism, MTHFR and Hepatoma

Different forms of folate cofactors are required in the reactions of DNA synthesis and methyl donor supply. Perturbations in folate-dependent methylation pathways have been associated with cancer occurrence [3]. Folate-mediated one-carbon metabolisms have been an important therapeutic target for numerous human diseases [14,15], including HCC [4]. However, the understanding of the repercussions of folate deficiency or supplementation on folate metabolic gene variations in the liver is limited.

Methylenetetrahydrofolate reductase (MTHFR, EC 1.7.99.5) is the enzyme catalyzing the irreversible conversion of 5,10-methylenetetrahydrofolate (methyleneTHF, 5,10-CH_2_-THF) to 5-methyltetrahydrofolate (methylTHF, 5-CH_3_THF) that can be inhibited by the universal methyl donor *S*-adenosylmethionine (SAM) [16]. By controlling folate cofactor distributions, MTHFR can modulate the partitioning of intracellular one-carbon moieties among purine synthesis, thymidine synthesis, and methyl donor supply [5].

Mildly reduced MTHFR is common in many populations due to a polymorphism at bp 677 known as C677T. This common mutation results in a thermolabile variant of the MTHFR enzyme with reduced activity in vitro [17] and an altered folate cofactor distribution in red blood cells [18]. The association between MTHFR polymorphisms and HCC risk is inconsistent and remains controversial [19]. In 2013, a meta-analysis paper reported that MTHFR A1298C polymorphism may play a protective role in the carcinogenesis of HCC [20]. Another meta-analysis paper reported in 2014 that MTHFR C677T polymorphism was significantly associated with an increased HCC risk in Asians but not in Caucasians [21], and in 2015 another meta-analysis also reported that MTHFR A1298C polymorphism might be related to an increased risk of HCC in Asians [22].

### 1.3. Folate Co-Factors vs. the Balance between Methyl Group and Nucleotide Supplies

Previous studies indicated that folate cofactors are limited for cytoplasmic folate-dependent reactions and that the synthesis of DNA precursors competes with SAM synthesis [23]. Due to distinct expression patterns of tissue-specific metabolic enzymes, metabolic kinetics in response to medications [5,24,25] or under nutritional deprivation [26] may differ among cell types. We previously demonstrated that MTHFR C677T polymorphism increases methotrexate (MTX) sensitivity via the inhibition of SAM and de novo purine synthesis [5]. We also demonstrated that lymphoblast is more sensitive to antifolate drug MTX in cell proliferation, protein, and thymidine synthesis, yet HepG2 is more sensitive in SAM supply. The MTHFR function affects purine but not thymidine synthesis in the MTX treated lymphoblast model [5]. Previous studies suggested that genetic predisposition, including MTHFR, could impact tumorigenesis in a tissue-specific manner [27], and these impacts could be specific to the pathway and closely related to nutritional factors including folate [28]. Variations in the MTHFR gene are related to alterations in folate form distributions. How such alterations impact 5-methylTHF dependent biochemical pathways such as SAM supply under folate depletion is unclear. In the plasma of women who received primed, constant infusions of isotopic tracer, TT genotype had elevated homocysteine synthesis compared to CC, but total remethylation was unchanged by the MTHFR 677C->T polymorphism [29]. In human monocytes, MTHFR TT genotype had marginally higher thymidylate synthesis than CC subjects, but purine synthesis was not affected by MTHFR genotype or folate depletion [30]. How MTHFR genetic variation affects liver transmethylation and nucleotide supply has not been fully elucidated. Therefore, well-designed and rigorously controlled studies are needed to investigate the impacts of MHTFR in more depth. Folate deficiency in mammals may cause an imbalance in the deoxynucleotide precursors for DNA replication/repair that affects the fidelity of DNA synthesis and predisposes to uracil misincorporation and DNA repair-related DNA strand breaks [31]. Dietary folate deficiency has been shown to cause progressive DNA strand breaks within exons 5–8 of the p53 gene in the rat colon [32], and also results in uracil accumulation in mouse liver DNA [8].

### 1.4. MTHFR and DNA Stability

Antisense inhibition of MTHFR reduces the survival of methionine-dependent tumor lines derived from colon, lung, breast, prostate, and neuroblastoma tumor cells [33,34]. As MTHFR inhibition decreases tumor growth, it was suggested that inhibition of MTHFR may be a potential anticancer approach. Low dietary folate and MTHFR deficiency reduce adenoma formation in mice predisposed to tumorigenesis, possibly through increased apoptosis consequent to hyperhomocysteinemia and nucleotide imbalances [35].

The impacts of MTHFR 677T polymorphism appear to be closely related to folate status. We have demonstrated that human lymphoblasts with MTHFR TT genotype had significantly reduced folate-dependent remethylation under folate restriction but had increased purine synthesis when folate was abundant compared to the TT genotype, presumably via increased formylated folate pool [26]. We suggest that the advantage of de novo purine synthesis found in the MTHFR TT genotype may account for the protective effect of MTHFR in hematological malignancies.

However, the impacts of MTHFR on hepatic tissues could be different. The impacts of MTHFR function and its interactions with folate status on hepatic SAM homeostasis, nucleotide supplies, and DNA integrity are complex and difficult to investigate due to the limited availability of hepatic tissues and various dietary folate consumptions in humans. The understanding of folate metabolic gene variations, hepatic folate deficiency, and liver chromosome stability need to be further elucidated. An in vitro cell model of liver origin would be feasible in studying the gene-nutrient interactions in this regard. The present study established HepG2 cell models with stabilized inhibition of MTHFR using small hairpin RNA delivered by a Lentiviral vector, and characterized the impacts of reduced MTHFR and folate status on cell populations, SAM homeostasis, nucleotide biosynthesis, and DNA stability, all of which are pathways involved in DNA integrity and repair that are critical in human tumorigenesis. This study provided insight into the significant impact of MTHFR function on chromosome stability of hepatic tissues. Data from the present study may shed light on the potential regulatory mechanism by which MTHFR modulates the risk for hepatic malignancies.

## 2. Results

### 2.1. Lower MTHFR Gene Expression Increased Survival in HCC Patients

**Figure 1:**
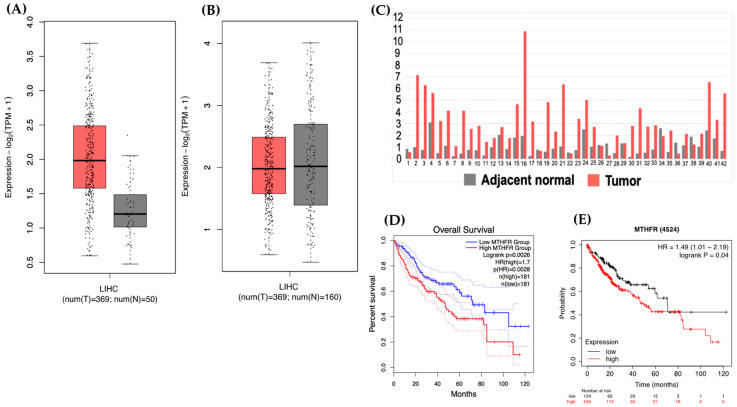
Reduced survival in patients whose tumor had higher MTHFR mRNA expression. (**A**) Box plots of MTHFR mRNA expression in HCC tissues (red) and the normal data from the TCGA database (gray); (**B**) Box plots of MTHFR expression of HCC tissue (red) compared to the normal data from the TCGA plus GTEx (gray); (**C**) Bar graph of MTHFR gene expression in paired HCC tumor and tumor-adjacent normal tissues from the Cancer RNA-Seq Nexus. The *X*-axis indicates the anonymous patient ID (*n* = 42); and the *Y*-axis indicates the fold change of MTHFR mRNA expression level (fragments per kilobase per million, FPKM) in the HCC (red) compared to those in the matched adjacent normal tissues (gray). Thirty-three out of the 42 HCC tissues had MTHFR overexpression in the tumor compared to its paired normal tissue; (**D**) Kaplan–Meier plots were generated from the Pan-cancer RNA-Seq Web server (**D**) and the GEPIA website (**E**). Both plots suggested that an increased MTHFR mRNA was significantly associated with poorer survival in HCC patients. Footnotes: The Cancer Genome Atlas (TCGA) is a landmark cancer genomics program that molecularly characterized over 20,000 primary cancers and matched normal samples spanning 33 cancer types. The Genotype-Tissue Expression (GTEx) project is an ongoing effort to build a comprehensive public resource to study tissue-specific gene expression and regulation. Samples were collected from 54 non-diseased tissue sites across nearly 1000 individuals, primarily for molecular assays including RNA-Seq. We compared MTHFR mRNA expression in HCC tissues to the normal tissue data from the TCGA and GTEx.

We aimed to compare the MTHFR mRNA expression in HCC patients and explored whether it is associated with HCC prognosis. MTHFR mRNA expression and its association with HCC survival were investigated by the GEPIA web tool on 14 May 2021. (http://gepia2.cancer-pku.cn/#index). The median of MTHFR mRNA expression level in HCC tissues tended to be elevated when compared to those normal data from The Cancer Genome Atlas (TCGA) (*n* = 389 for tumor and *n* = 50 for controls, Figure 1A). When we compared the MTHFR gene expression of HCC to the normal data from the TCGA combining and from The Genotype-Tissue Expression project (GTEx), no difference was found (Figure 1B). We supposed that there could be systemic variations in gene expression profiles between different studies and databases, therefore, we further compared the MTHFR gene expression in a RNA-Seq dataset containing 42 paired tumor and tumor-adjacent normal HCC tissues from the Cancer RNA-Seq Nexus on 14 May 2021. (CRN, http://syslab4.nchu.edu.tw/CRN) [36,37]. Thirty-three out of the 42 HCCs had MTHFR overexpression in the tumor compared to its paired normal tissues (Figure 1C). The mean MTHFR gene expression level (fragments per kilobase per million, FPKM) in tumor tissues were approximately threefold of that in the adjacent normal tissues (*p* = 3.88777 × 10^−8^).

The survival analysis was performed using the Pan-cancer RNA-Seq Web server (http://kmplot.com/analysis/index.php?p=service&cancer=liver_rnaseq, acessed on 14 May 2021). The Kaplan–Meier (K–M) plots were generated by auto-selecting the best cutoff values between lower and upper quartiles into high and low expression groups that included all stages, sex, race, and mutation burden [38] (Figure 1D). A K–M plot was also generated from the GEPIA website (Figure 1E). Higher MTHFR mRNA expression was significantly associated with poorer survival in HCC patients in both K–M plots (*p* = 0.04 and *p* = 0.0028, respectively).

Taken together, compared to HCC tissues, MTHFR mRNA expression was lowered in the adjacent non-tumor tissues. Furthermore, lower MTHFR gene expression in the tumor tissues increased the survival chance in HCC patients. These results suggested a potential survival advantage of lower *MTHFR* gene expression in HCC.

### 2.2. shRNA Lentivirus Production and Transfection in HEK 293T Cells

To further investigate how *MTHFR* expression affects cell function and metabolism, stable *MTHFR* knockdown HepG2 cells were established using the lentiviral small hairpin RNA (shRNA) interference vectors described in detail in the Materials and Methods Section. Stable cell lines were established from the human hepatoma cell-line HepG2 that retains morphological, and biochemical characteristics of normal human hepatocytes [8].

The short hairpin RNA (shRNA) constructs were based on the pLKO.1-puro vector. These lentivirus-based shRNA constructs were obtained from the National RNAi Core Facility (RNA technology platform and gene manipulation core, Academia Sinica, Taiwan). The target sequences of various shRNAs are listed in Table 1A and their target sites are provided in Supplementary Figure S1. Different shRNA lentiviruses were produced from HEK293T packaging cells that were transfected separately with either MTHFR shRNA (sh3′UTR, sh77, sh546, sh697, sh1618) or empty EGFP lentiviral plasmids. To do so, virus-producer human embryonic kidney cell line 293T (HEK 293T) cells were seeded 1 day before transfection and then transfected with the above plasmid DNAs. The transfected GFP-expressing HEK293T cells were examined under a fluorescence microscope (Supplemental Figure S2A–C), and the GFP fluorescence intensity were measured by flow cytometry (Supplemental Figure S2D). The number of GFP-positive cells (IU: infectious unit) was divided by the volume of viral solution (mL) to calculate virus titer. The flow cytometry results of the empty lentiviral EGFP vector showed an estimation of 99% transfection efficiency (Supplemental Figure S2D), proving that a successful HEK 293T packaging line was established using the lentiviral system. The supernatants from different virus-producing HEK293T cells were collected 48 h post-transfection that were then used to infect HepG2 cells (more details are described in the Materials and Methods section). Forty-eight hours after the virus infection, the transduced HepG2 cells were selected for individual stable clones using puromycin (20 μg/mL) for at least 2 weeks; then the surviving cells were cultivated and maintained for further applications.

### 2.3. Efficiency of Different Target shRNA Sequences on MTHFR mRNA Reduction

**Figure 2:**
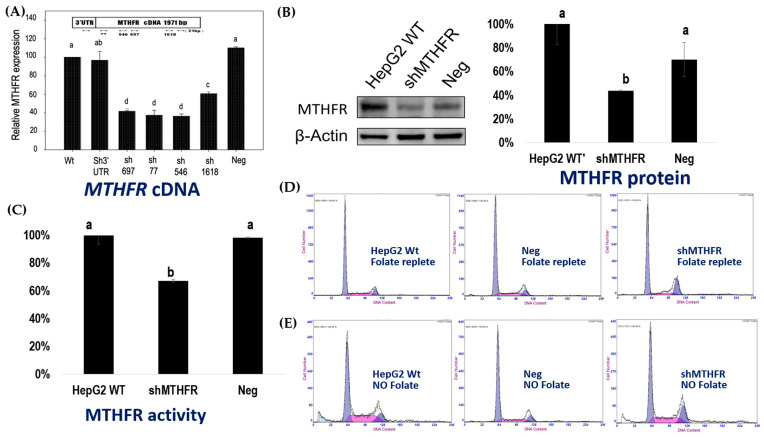
Stabilized inhibition of MTHFR in HepG2 cells using RNAi delivered by lentiviral vector. (**A**) Five different homologous human shRNA target sequences were designed for human MTHFR gene in order to study the impacts of MTHFR gene silencing. These RNAi lentivirus clones are termed sh3′UTR, sh77, sh546, sh697, sh1618 that represented the target site on the MTHFR cDNA sequence (see Supplemental Data File for Figure S1). The shGFP clone was used as the negative control cell-line (Neg). Among them, clone sh77 had a significant reduction (by ~63%) in MTHFR mRNA expression and was chosen to represent the shMTHFR cell line for further studies. (**B**) shMTHFR (sh77) had reduced MTHFR protein expression compared to WT and Neg cells. Data are shown as mean with SE for triplicate samples. Different letters indicate statistical differences (*p* < 0.05) among different groups calculated by one-way ANOVA). (**C**) shMTHFR (sh77) cells had reduced MTHFR enzyme activity compared to WT and Neg cells. MTHFR enzyme activity was determined by radioisotope analysis described in the Materials and Methods section. Data are shown as mean ± SE for triplicate samples. Different letters indicate statistical differences (*p* < 0.05) among different groups calculated by one-way ANOVA). (**D**) shMTHFR significantly decreased cell populations in the G1 and S phases and increased that in the G2/M phase in adequate folate. The quantitative data for flow cytometry are shown in Table 1B. (**E**) The impacts of MTHFR and folate depletion on cell cycle distributions. The quantitative data for flow cytometry are shown in Table 1B. The original flow cytometry data presented in Table 1B can be found in Supplemental Figure S3.

Five different homologous human shRNA target sequences were designed for the human MTHFR gene in order to study the impacts of MTHFR gene silencing. These RNAi lentivirus clones are termed (1) sh3′UTR, (2) sh77, (3) sh546, (4) sh697, (5) sh1618 that represented their target sites on the MTHFR cDNA sequence (Table 1A and Supplemental Figure S1). The shGFP clone was used as the negative control cell-line (Neg) as it underwent the same lentiviral transfection procedure but it did not target a specific human gene sequence. The clone was transduced by a GFP shRNA-expressing plasmid as a non-silencing shRNA construct (pLKO.1-shGFP). Relative efficiency of different target shRNA sequences on the reduction of MTHFR expression was determined by real-time PCR (Figure 2A) and their relative expression compared to wildtype (WT, as 100%) are shown in Table 1B: (1) sh3′UTR: 96% ± 0.10; (2) sh77: 37 ± 0.06%; (3) sh546: 44 ± 0.03%; (4) sh697, 42 ± 0.01%; (5) sh1618: 61 ± 0.02% and (6) Neg 110 ± 0.01%. The effects of various shMTHFR on cell cycle distributions were further investigated. Clone sh77 had a significant reduction (by ~63%) in MTHFR mRNA expression, and it was chosen to represent shMTHFR for further experiments. shMTHFR (sh77) had reduced MTHFR protein expression (Figure 2B) and reduced MTHFR enzyme activity (Figure 2C) compared to WT and Neg cells.

### 2.4. Lower MTHFR Is Associated with Decreased Proportional Cell Populations in the G1 Phase and an Increase in Those in the G2/M Phase

shMTHFR significantly decreased cell populations in the G1 and S phases and increased those in the G2/M phase (Figure 2D and Supplemental Figure S4). The quantitative data of cell cycles in adequate folate conditions are shown in Table 1B. Relative MTHFR mRNA expression and their associations between MTHFR gene expression and cell cycle distributions are shown in Table 1C Interference of MTHFR expression by shMTHFR significantly decreased cell populations in the G1 and S phases and increased that in the G2/M phase. *Pearson’s* correlation analysis among these cell lines indicated that the G1 phase cell population significantly correlated with MTHFR gene expression (r = 0.86; *p* < 0.0001), and that the G2/M phase cell population inversely correlated with MTHFR gene expression (r = −0.808; *p* < 0.0001; Table 1D. No correlation was found between the cell population in the S phase with the MTHFR gene expression.

WT, sh77 that had a 63% reduction in MTHFR mRNA, and the negative control cell lines (Neg) were used in further metabolic experiments, and the associations between cell cycle distribution with MTHFR protein and enzyme activity were examined in these cell-lines. Cell population in the G2/M phase appeared to be closely related to MTHFR function, as they highly inversely correlated with MTHFR gene level (r = −0.81, *p* < 0.0001), MTHFR protein expression (r = −0.8; *p* = 0.01), and MTHFR enzyme activity (r = −0.842; *p* = 0.005) (Table 1D).

### 2.5. Reduced MTHFR Is Associated with Decreased Intracellular SAM and SAH Contents

Tetrahydrofolate (THF) is a metabolic cofactor that carries and activates single carbons for the synthesis of purine and thymidine nucleotides [39] and homocysteine remethylation to methionine [40]. Folate-mediated one-carbon metabolism is compartmentalized in the mitochondria, nucleus, and cytoplasm of eukaryotic cells [41].

Impaired folate-dependent methionine synthesis can reduce cellular methyl donor SAM. Perturbations in SAM homeostasis impair methylation reactions that may alter gene expression and genome stability. In our knock-down cell-line shMTHFR (sh77) cultured in adequate folate media, knocking down MTHFR significantly decreased cellular SAM, and SAM to *S*-adenosylhomocysteine (SAH) ratio in HepG2 cells. Compared to the WT and Neg cells, shMTHFR 77 decreased intracellular SAM by 31 and 42%, respectively (Table 2A). *Pearson’s* correlation analysis among these cell lines indicated that SAM contents significantly correlated with MTHFR enzyme activity (r = 0.868; *p* < 0.003) (Table 2B).

### 2.6. shMTHFR Did Not Intensify Low Folate Induced SAM Reduction in HepG2 Cells

In adequate folate, shMTHFR drastically increased the proportion (from 9 to 22%) of cell populations in the G2/M phase, which was found to be highly correlated with the mRNA level, protein abundance, and the enzyme activity of MTHFR. shMTHFR also significantly decreased SAM content that was significantly correlated with MTHFR enzyme activity. Previously we demonstrated that the impacts of genetic variations in MTHFR are closely related to folate status [26,27], and folate depletion has been reported to alter cell cycle distributions [42] and SAM synthesis [26]. Therefore, we further investigated whether shMTHFR may intensify its impacts on G2/M cell population distribution and SAM contents under folate depletion.

Folate depletion modestly but significantly increased the G2/M populations in both WT and Neg HepG2 cells. However, in contrast to WT and Neg cells that had increased G2/M cell populations in folate depletion, the proportional G2/M populations in shMTHFR cells significantly decreased in response to folate depletion (Figure 2E, Table 3A). These results indicated that shMTHFR did not augment folate deficiency induced G2/M cell cycle arrest in HepG2 cells, and that shMTHFR somewhat appeared to alleviate the impacts of folate depletion on cell cycle distribution. When cells were depleted with folate and then cultured in low folate, SAM contents did not differ among WT, Neg, and shMTHFR under the low folate condition (Table 3B). Compared to cells cultured in folate-repletion, such a low folate condition decreased SAM in WT, Neg and shMTHFR by 28.6, 44, and 19.9%, respectively. The relatively fewer SAM reductions in shMTHFR in low folate indicated that shMTHFR does not intensify folate depletion-induced methyl group supply. On the other hand, shMTHFR tended to increase SAH level (*p* < 0.2) and significantly decreased the SAM to SAH ratio in low folate conditions. The accumulation of SAH may suggest that shMTHFR cells are more sensitive to folate depletion with respect to homocysteine remethylation. Whether shMTHFR impacts chromosome stability during folate depletion was investigated further.

### 2.7. shMTHFR Promoted Nuclei SHMT1/DHFR/TYMS Protein Expression under Folate Deficiency

Deoxynucleoside triphosphates (dNTPs) are essential for the replication and maintenance of genome stability. The de novo thymidylate biosynthetic pathway in mammalian cells has a multienzyme complex consisting of serine hydroxymethyltransferase 1 and 2α (SHMT1 and SHMT2α), thymidylate synthase (TS), and dihydrofolate reductase (DHFR) that translocates to the nucleus for DNA replication and repair [43]. The nuclear localization of SHMT1, TYMS and DHFR have been determined as a function of the cell cycle. Co-localization of DHFR and SHMT1 with lamin B1 is concomitant with nuclear localization of DHFR and SHMT1 during S and G_2_/M, but not during the G_1_ phase of the cell cycle [43].

**Figure 3:**
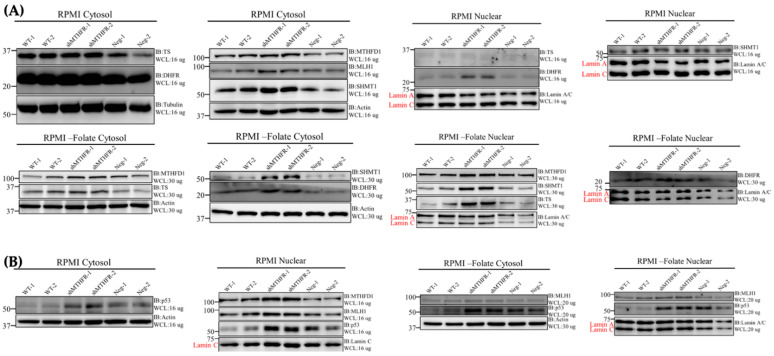
shMTHFR enhanced cytosolic and nuclear SHMT/DHFR/TYMS protein complex, MTHFD1 (**A**), as well as DNA repair protein MLH1, and p53 expressions (**B**) in folate repletion and folate depletion. The quantifications of SHMT/DHFR/TYMS and MTHFD1 are shown in Table 4. The total abundance of the SHMT/DHFR/TYMS protein complex (combined cytosol and nucleus) significantly increased by shMTHFR, especially under folate depletion. Folate depletion induced MTHFD1 expression in shMTHFR and such induction was more drastic in the nucleus in response to folate depletion (Table 4B). These results may account in part for the stable isotopic tracer experiments using L- [3-^13^C]-serine. After cells were initially depleted of folate and then supplemented with low dose folinate, deoxythymidine monophosphate (dTMP) enrichments (dT+1 represents M+1 of dTMP) from [3-^13^C]-serine were significantly higher in shMTHFR compared to Neg and WT (Table 5). In addition, shMTHFR induced hMLH1 and p53 expression in both folate depletion and repletion, consisting with the finding of reduced DNA instability (Table 6A,B) in shMTHFR. The quantifications of MLH1 and p53 protein expression are shown in Table 6C. Moreover, folate restriction decreased nuclear and cytosol hMLH1 and p53 protein abundance, and shMTHFR recovered the reduction of hMLH1 and p53, especially in the nucleus (Figure 3B, Table 6C). Abbreviations: SHMT1, serine hydroxymethyl-transferase1; TS, thymidylate synthase; DHFR, dihydrofolate reductase; MTHFD1, methylenetetrahydrofolate dehydrogenase; hMLH1, human Mut L homologue-1.

As shMTHFR was discovered to induce cell G2/M arrest in HepG2 cells, we speculated that it may assist de novo thymidylate synthesis via the induction of this multienzyme complex. Effects of shMTHFR and folate supply on cytosolic and nucleus SHMT1, TYMS and DHFR were determined in WT, Neg and shMTHFR HepG2 cells under folate repletion or folate depletion. The total abundance of all above proteins (combined cytosol and nucleus) significantly increased by shMTHFR, especially under folate depletion. In the cytosolic fraction, folate depletion resulted in a drastic decrease in SHMT1, DHFR, TYMS protein abundance compared to those cultured in folate repletion (Figure 3A, Table 4A). In response to folate depletion, the mean abundance of cytosolic SHMT1 decreased by 75, 14, and 68% in WT, shMTHFR, and Neg, respectively. Cytosolic DHFR decreased by 28, 17, and 71% in WT, shMTHFR, and Neg, respectively. Cytosolic TYMS decreased by 37, 35, and 48% in WT, shMTHFR, and Neg, respectively.

In contrast to cytosol SHMT1/DHFR/TYMS proteins that drastically decreased in response to folate depletion, the SHMT1/DHFR/TYMS proteins appeared to significantly increase in the nucleus during folate depletion (Figure 3A, Table 4B). In particular, the increase in SHMT1/DHFR/TYMS in the nucleus was more drastic in the shMTHFR in response to folate depletion. These results suggest that shMTHFR may protect the thymidine synthetic pathway during folate depletion by promoting the translocation of the SHMT1/DHFR/TYMS complex.

In the present study, folate depletion significantly decreased cytosol and nuclear methylenetetrahydrofolate dehydrogenase (MTHFD1) abundance. Yet, shMTHFR cells had significantly higher MTHFD1 abundance in both cytosol and nucleus under both folate repletion and depletion (Figure 3A, Table 4). MTHFD1 was reported to regulate nuclear de novo thymidylate biosynthesis and genome stability [44]. In particular, nuclear enrichment of folate cofactors and MTHFD1 can protect de novo thymidylate biosynthesis during folate deficiency [44]. Under folate depletion, compared to WT and Neg, shMTHFR increased nucleus MTHFD1 expression by 64 and 79%, respectively.

We postulate that by promoting the abundance and translocation of the SHMT1/DHFR/TYMS complex as well as nucleus MTHFD1 abundance during folate depletion, shMTHFR can help preserve the nucleotide pool that may protect against chromosome instability. The impacts of shMTHFR on nucleotide biosynthesis and chromosome stability under depletion were studied further.

### 2.8. shMTHFR Assisted Purine Synthesis in HepG2 Cells under Folate Deficiency; Such Impacts Were Amplified after Folinate Supplementation

Folate depletion and folinate supplementation change the partitioning of 5,10-CH_2_THF dependent 1C metabolic fluxes between mitochondrial and cytosolic derived formate in vivo [45]. It is plausible that MTHFR can regulate the competition between folate-dependent deoxyribonucleotide and SAM biosynthesis by controlling the balance between folate cofactor methyleneTHF (5,10-CH_2_THF) and methylTHF (5-CH_3_THF) [26]. Since reduced MTHFR may assist in shuttling more cytosolic 5,10-CH_2_THF for nucleotide biosynthesis [26], we then investigated how shMTHFR affects 1C metabolic fluxes in nucleotide biosynthesis under folate restriction using L-[3-^13^C]-serine.

In this experiment, cells were either cultured in folate depletion, or depleted with folate and then supplemented with low levels of folinate. Enrichments in the nucleotides from L- [3-^13^C]-serine were compared between WT, Neg, and shMTHFR cells in folate depletion and the folate deplete/replete condition. After cells were initially depleted of folate and then supplemented with low dose folinate, deoxythymidine monophosphate (dTMP) enrichments (dT+1 represents M+1 of dTMP) from [3-^13^C]-serine were significantly higher in shMTHFR compared to Neg and WT (Table 5); dT+1 enrichments appeared to be similar among these cell-lines when cultured in folate repletion or no folate.

Purine synthesis via cytosolic 10-formyl-THF pool was assessed by the mass isotopomer analysis (MIA) as previously described [23,46] and the purine enrichments from [3-^13^C]-serine. Folate depletion significantly decreased the enrichments and MIA of purines (deoxyadenosine, or dA, and deoxyguanosine, or dG) in all cell-lines. Under folate repletion, WT, Neg, and shMTHFR cells had similar MIA. It is noteworthy that shMTHFR had less reduction compared to that in WT and Neg cells in response to folate depletion (Table 5). The impacts of folate depletion/low folate seemed milder in shMTHFR compared to WT and Neg. Folinate has been shown to be effective in rescuing certain impaired 1C metabolic pathways induced by MTX [45] or folate depletion in vivo [47,48]. In the present study, shMTHFR seemed to have better rescuing effects from folinate after initial folate depletion in our cell model. These data indicate that shMTHFR may assist the adaptation of folate depletion by augmenting purine supply, and it is plausible that shMTHFR may protect cells against folate depletion induced chromosome instability.

### 2.9. shMTHFR Reduced Micronucleated Binucleated Cells and Uracil Misincorporation into DNA under Folate Deficiency

Folate depletion causes chromosome instability by increasing DNA strand breakage, uracil misincorporation, and defective repair. Human lymphocytes cultured in folate-deficient media exhibit DNA double strand breaks [9], reduced DNA repair [9], and micronuclei formation [10,11,12]. High dietary folate consumption is associated with lower micronucleus frequency in humans [13].

In the present study, folate depletion increased the G2/M populations in both WT and Neg but not in shMTHFR HepG2 cells. These findings implied that shMTHFR does not exacerbate the cell cycle arrest in G2/M, but rather may alleviate the impacts of folate deficiency. As micronuclei formation is generally attributed to an error in DNA synthesis during the S phase or in mitosis during the G2/M phase in the cell cycle [49], we speculated that shMTHFR may also help to minimize micronuclei and protect DNA stability during folate depletion by prolonging the G2/M phase for DNA repair. Our results supported the above postulation. shMTHFR had significantly fewer micronuclei regardless of folate status. Compared to WT, shMTHFR reduced the number of micronuclei by 63, 52, and 73% in folate repletion, low folate, and folate depletion, respectively. Similarly, compared to Neg, shMTHFR reduced the number of micronuclei by 76, 62, and 58% in folate repletion, low folate, and folate depletion, respectively (Table 6A).

Regulation of cellular dTMP synthesis is essential for DNA replication and genome stability in the nucleus [6]. Impaired de novo thymidylate synthesis due to folate deficiency results in deoxyuridine/uracil misincorporation into DNA and causes genome instability [7]. In the present study, we further discovered that shMTHFR promoted the expression and translocation of SHMT1/DHFR/TYMS complex in the nucleus during folate deficiency. Since nuclear localization of de novo thymidylate biosynthesis pathway is required to prevent uracil accumulation in DNA [50], it is likely that shMTHFR can help reduce folate deficiency induced uracil accumulation and protect DNA.

Compared to WT and Neg, shMTHFR reduced uracil contents by 7.44 and 6.97%, respectively in the folate repletion (Table 6B), supporting our postulation. Moreover, shMTHFR significantly reduced uracil contents in DNA by 16.2 and 16.9% compared to WT and Neg, respectively, in folate depletion (Table 6B). These data again suggest that shMTHFR is protective against folate depletion induced chromosome instability.

### 2.10. shMTHFR Promoted Nuclear MLH1/p53 Expression under Folate Deficiency

It has been suggested that agents that induce DNA mispairs will cause G2 arrest in mismatch repair-proficient cells [51]. Since we discovered that shMTHFR induced cell cycle arrest in the G2/M phase, we aimed to investigate whether shMTHFR may protect cells from folate depletion-induced DNA damage. DNA repair processes are critical mediators of p53-dependent tumor suppression [52], and p53 has been found to be required for the folate depletion-induced apoptosis process. Furthermore, Human Mut L homologue-1 (hMLH1) is one of the key proteins involved in the mismatch repair process after DNA replication, and defected hMLH1 is commonly found in moderately and poorly differentiated hepatocellular carcinoma [53]. It has been reported that cells proficient in mismatch repair were highly sensitive to folate deficiency compared with cells defective in mismatch repair proteins [54], and in vitro biochemical studies demonstrated a direct participation of mismatch repair proteins in mediating the apoptotic response induced by folate deficiency [54].

Our study demonstrated that shMTHFR induced hMLH1 and p53 expression in both folate depletion and repletion, consistent with the finding of reduced micronucleated/binucleated cells and uracil misincorporation (Table 6C). Moreover, folate restriction decreased nuclear and cytosol hMLH1, p53 protein abundance, and shMTHFR recovered the reduction of hMLH1, p53 (Table 6C).

## 3. Discussion

**Figure 4:**
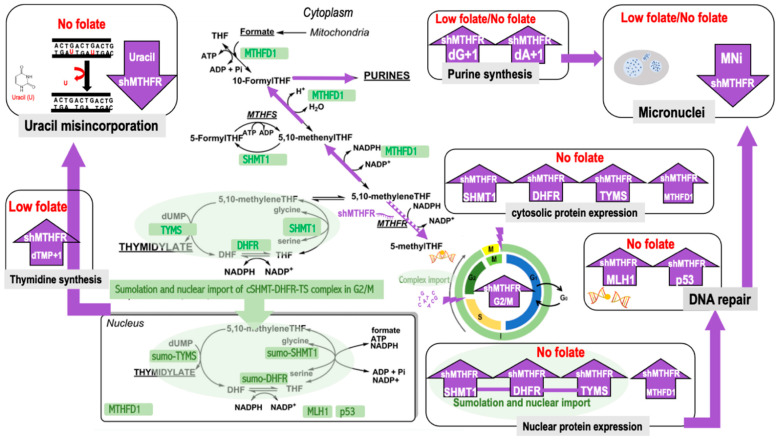
MTHFR knockdown by shRNA can protect DNA under folate deficiency. Lower MTHFR is associated with increased proportional cell populations in the G2/M phase, increased cytosol, and nuclear SHMT1/DHFR/TYMS protein expression under folate deficiency. shMTHFR assisted purine synthesis in HepG2 cells under folate deficiency and such impacts were amplified after folinate supplementation. shMTHFR promoted nuclear MLH1/p53 expression under folate deficiency that can protect cells from folate depletion induced micronuclei and uracil misincorporation. Abbreviations: MTHFD1, methylenetetrahydrofolate dehydrogenase; SHMT, serine hydroxymethyltransferase; MTHFR, methylenetetrahydrofolate reductase; GNMT, glycine N-methyltransferase; MTR, methionine synthase; MAT, *S*-adenosylmethionine synthase; SAHH, S-adenosylhomocysteinehydrolase; TS, thymidylate synthase; DHFR, dihydrofolate reductase; THF, tetrahydrofolate; dUMP, deoxyuridine monophosphate; DHF, dihydrofolate; hMLH1: Human Mut L homologue-1 (hMLH1).

The present study demonstrated that MTHFR knockdown in liver-origin cells may assist the defense against folate depletion-induced chromosome segregation and uracil misincorporation in the DNA (Figure 4).

To better understand the interactions between MTHFR function and chromosome stability under different folate supplies, we successfully established in vitro cell models of liver origin with stabilized inhibition of MTHFR using small hairpin RNA. The cell model was then characterized with respect to cell cycle, SAM homeostasis, folate-dependent de novo nucleotide biosynthesis, and DNA stability under folate depletion.

In our cell model, reduced MTHFR function is associated with decreased cell populations in the G1 phase and increased cell populations in the G2/M phase. The proportion of cell population in the G2/M phase was highly inversely correlated with MTHFR gene level, protein abundance, and enzyme activity, suggesting that MTHFR function is closely related to the cellular events during G2/M.

The impacts of MTHFR function have been reported to be closely related to folate status. We previously demonstrated that human lymphoblasts with MTHFR TT genotype had significantly reduced folate-dependent remethylation and SAM contents when folate supply was restricted. In folate restriction, the reduction of SAM supply in TT genotype was threefold (decreased by 27%) of that in the CC genotype (decreased by 9%) [26]. We also demonstrated an increase in purine synthesis in the TT lymphoblasts compared to that of the CC lymphoblasts when folate was abundant, presumably due to the increased formylated folate pool [26]. We suggested that the advantage of de novo purine synthesis found in the MTHFR TT genotype may account for its protective effect in hematological malignancies. Methyl donor and/or folate deficiency is also associated with genomic damage and cell death in human lymphocytes in vitro [10]. Folate deficiency may deplete cellular SAM supply and perturb methylation reactions of DNA, RNA, and histones, leading to altered gene expression and genome stability. By catalyzing the irreversible conversion from 5,10-methyleneTHF to 5-methylTHF, MTHFR may regulate the competition between folate-dependent dTMP and SAM biosynthesis. However, genetic predisposition, including variations in MTHFR function, could impact folate mediated transmethylation and nucleotide synthesis in a tissue-specific manner [27]. MTHFR C677T mutation induces cell-specific changes in genomic DNA methylation and uracil misincorporation that could in part account for the molecular basis for the site-specific risk modification in tumors from different tissue origins [27]. Unlike the lymphoblast models with TT genotype that were more sensitive to folate depletion in SAM synthesis, shMTHFR did not intensify folate depletion induced G2/M cell cycle arrest or SAM reduction in our HepG2 cell model. These findings suggest that, unlike extrahepatic cells that have enhanced nucleotide biosynthesis at the cost of reductions in the methyl group supply during folate depletion, lower MTHFR in cells of liver origin may take advantage of the nucleotide biosynthesis without severe impacts on SAM supply, possibly due to other methyl sources such as choline (via the betaine homocysteine *S*-methyltransferase pathway).

shMTHFR appeared to facilitate HepG2 cells entering the S phase and prolong G2/M in the cell cycle. Knockdown of MTHFR has been shown to decrease gastric cancer cell survival and result in cell cycle arrest at the G2 phase [55]. The shMTHFR induced cell cycle arrest raised our interest as cells in G2/M undergo multiple processes involved in DNA damage and repair. DNA instability (strand breakage, uracil misincorporation, and defective repair) is increased by folic acid depletion in human lymphocytes in vitro [9].

The nuclear localization of SHMT/TS/DHFR multienzyme complex has been determined as a function of cell cycle that is required for of de novo thymidylate biosynthesis [43]. The nuclear translocation of SHMT1 is cell cycle-dependent and occurs during the S and G_2_/M phases [56,57]. DHFR and TYMS also localize to the nucleus during S and G_2_/M phases but not in the G_1_ phase. In the present study, folate depletion resulted in a significant reduction in SHMT/DHFR/TYMS, and shMTHFR-promoted nuclei SHMT1/DHFR/TYMS protein expression and translocation, in particular, under folate deficiency.

The reductive methylation of uridylate monophosphate (dUMP) to thymidylate monophosphate (dTMP) involves the transfer and, simultaneously, the reduction of the one-carbon moiety from 5,10-methyleneTHF. The 5,10-methyleneTHF is produced either by the activity of SHMT or by MTHFD1 [43]. Metabolic studies in MCF-7 cells demonstrated that SHMT contributes approximately 30% whereas MTHFD1 contributes 70% of one-carbon groups used in the reductive methylation of dUMP catalyzed by TYMS [23]. The THF is regenerated by the activity of DHFR [44]. In addition to the increased abundance of SHMT1/DHFR/TYMS proteins, shMTHFR also induced MTHFD1 expression both in the cytosol and nucleus in our HepG2 cell model.

Folate depletion impairs nucleotide biosynthesis that promotes DNA strand breakage, uracil misincorporation, and defective repair in human lymphocytes [9]. Reduced dTMP synthesis results in uracil incorporation into DNA. The DNA repair machinery can remove the uracil, but in the presence of a high deoxyuridine triphosphate (dUTP) to deoxythymidylate triphosphate (dTTP) ratio, it can be incorporated into DNA again. This futile cycle of uracil incorporation and deprived repair ultimately results in DNA double strand breaks and genome instability [9]. Unlike all other nucleotide synthesis that occurs in the cytoplasm, the de novo thymidylate biosynthesis pathway localizes to the nucleus and it is required to prevent uracil accumulation in DNA [50]. The nuclear compartmentation of de novo thymidylate biosynthesis at the replication fork enables the regulation of dUTP incorporation into DNA, as opposed to its misincorporation into DNA, for the regulation of transcription [43]. The present study provided direct evidence that by promoting the formation of the nuclear thymidylate biosynthesis multienzyme complex, shMTHFR effectively suppresses folate deficiency induced uracil accumulation thus helps protect DNA.

The rate of DNA synthesis is dependent on de novo purine synthesis. During the G1/S phase, the rates of purine synthesis via the *de novo* and the salvage pathway increased 5-fold and 3.3-fold, respectively, in human colon carcinoma cell HCT116 [58], indicating that when cells progress from mid-G1-phase to early S-phase, they must substantially increase the synthesis of purine nucleotides/deoxynucleotides. Defected purine synthesis due to folate depletion leads to chromosome instability, and reduced purine synthesis causes cytostasis and cytotoxicity, as well as aberrant DNA synthesis, repair, and mutagenesis [59,60]. Our study suggests that shMTHFR may assist cells passing the G1 and S phase and entering the G2/M stage more rapidly by shuffling more nucleotides. This may partially account for the observation that shMTHFR had more cell populations in the G2/M phase. Furthermore, the incorporations of serine derived one-carbon moiety into the purine ring were significantly increased by shMTHFR during folate depletion and such impacts were sustained after folinate supplementation followed by the initial folate depletion. These findings suggest that by preserving more one-carbon moieties in nucleotide biosynthesis, shMTHFR can help maintain cell cycle progression during starvation of metabolic intermediates, such as purines and pyrimidines, in this cell model. These data demonstrated an important role of MTHFR in the regulation of cell cycle progression, as well as the formation and translocation of the nuclear multi-enzyme complex during S and G2/M phases. Our study also suggested that MTHFR can assist nuclear de novo thymidylate synthesis during DNA replication and repair in folate deficiency.

In the present study, we discovered that shMTHFR promoted nuclear p53 (and MLH1) expression, especially under folate deficiency. p53 is the most commonly mutated gene in human cancer that acts as a major cell cycle checkpoint regulator. p53 is involved in various DNA-repair systems and takes on multiple mechanisms to prevent cancer development by maintaining genome stability [61]. p53 has been identified as a component of a spindle checkpoint that ensures the maintenance of diploidy [62].

Certain ribonucleotide biosynthesis inhibitors caused a p53-dependent G0 or early G1 arrest, and p53 was proposed to be a metabolite sensor activated by depletion of ribonucleotides or their related processes [63]. p53 was therefore suggested to play a role in inducing a quiescence-like arrest state in response to nutrient challenge and a senescence-like arrest state in response to DNA damage [63]. In normal human fibroblasts. CTP, GTP, or UTP depletion alone was sufficient to induce cell cycle arrest [63]. In the present study, when cells underwent folate depletion, the nucleotide shortage was less severe in cells with reduced MTHFR activity, and we postulate that the elevated p53 can help ameliorate the consequences of nucleotide depletion.

p53 was initially shown to act at the G1 checkpoint but was later also found to be important in regulating the spindle checkpoint during G2/M [62]. The spindle assembly checkpoint is a cell-cycle regulatory pathway preventing chromosomal instability. Prolonged folate depletion in human NCM460 colon mucosal cells substantially compromises the spindle assembly checkpoint network, which predisposes cells to mitotic aberrations and chromosomal instability [64]. Our results may suggest that shMTHFR can facilitate cell cycle progression and induce p53 expression that may effectively ameliorate folate depletion-induced mitotic aberrations and chromosomal instability, including impaired spindle assembly.

Folate adequacy protects against mutagenesis at the phosphatidylinositol glycan class A gene (Pig-a) locus and micronuclei induction in the red blood cells of mice [1]. Mice fed a folate-deficient diet had 1.8-fold higher micronuclei (MNi) in reticulocytes, and 1.5-fold higher micronuclei in normochromic erythrocytes than mice fed the FA supplemented diet [1]. Since micronuclei formation is generally attributed to error in DNA synthesis during the S and/or mitosis during G2/M phase in the cell-cycle [49], it is plausible that the prolonged G2/M resulted in shMTHFR cells may assist nucleotide supply and reduce DNA damage by repairing replication errors during folate depletion. shMTHFR significantly decreased folate depletion-induced micronuclei, supporting our hypothesis.

Cells proficient in mismatch repair were highly sensitive to folate deficiency compared with cells defective in mismatch repair proteins [54]. In vitro biochemical studies demonstrated a direct participation of mismatch repair proteins in mediating the apoptotic response induced by folate deficiency [54]. p53 plays diverse roles to directly impact the activity of DNA-repair systems and protects cells from cancer development by maintaining genome stability [61]. p53 was found to be required for the folate depletion-induced apoptosis process.

Loss of the DNA repair gene human Mut L homologue-1 (*hMLH1*) was found to cause lymphoma, and the enforced expression of *MLH1* was found to delay tumor development driven by loss of p53 [52]. These studies indicated that MLH1 mediated DNA repair processes are critical mediators of p53-dependent tumor suppression [52]. The impact of shMTHFR on hMLH1 in HCC has not been studied before.

hMLH1 is one of the key proteins involved in the mismatch repair process after DNA replication. Defected hMLH1 and hMSH2 commonly occur in moderately and poorly differentiated HCC [53]. Although it was suggested that defective DNA mismatch repair does not contribute greatly to hepatocellular carcinogenesis [65], combined loss of expression of O6-methylguanine-DNA methyltransferase and hMLH1have been reported to accelerate the progression of HCC [66]. Since we discovered that shMTHFR induced cell cycle arrest in the G2/M phase and promoted p53 expression, it is plausible that shMTHFR may protect cells from folate depletion-induced DNA damage via DNA repair process involved MLH1.

We found that shMTHFR not only promoted p53 but also induced hMLH1 expression in both folate depletion and repletion, consisting with the finding of reduced micronucleated binucleated cells and uracil misincorporation (Table 6C). In addition, folate restriction decreased nuclear and cytosol hMLH1 and p53 protein abundance, and shMTHFR recovered the reduction of hMLH1, p53 (Table 6C). These observations support our postulation that shMTHFR protects cells from folate depletion-induced DNA damage via p53 mediated MLH1 DNA repair.

DNA mismatch repair proteins MLH1 and PMS2 have been identified as p53 targets that may serve as a sensor in DNA repair mechanisms and a critical determinant for the decision between cell-cycle arrest and apoptosis [67]. Both of the *hMLH1* and *hPMS2* genes were determined to be responsive to DNA damage and p53 activation in normal human fibroblasts, and have p53-response elements within their first intron [67]. Deficiencies of DNA mismatch repair-complex proteins, including hMLH1 and hPMS2, typically result in microsatellite instability in human cancers [68]. A normal hMLH1 protein level is important in maintaining normal levels of hPMS1 and hPMS2 proteins. Gastric and colorectal cancer cells lines with microsatellite instability lacked detectable hMLH1, and the decreased hMLH1 has been associated with markedly reduced hPMS2 and hPMS1 proteins [68]. Yeast two-hybrid assay has been used to identify the amino acid residues in hPMS2 that interact strongly with hMLH1 [69].

On the other hand, it was reported that the concomitant loss of Pms2 function chemosensitises p53-deficient cells to some types of anticancer agents; Pms2 positively modulates cell survival by mechanisms independent of p53, and that increased cytotoxicity is paralleled by increased apoptosis. Tumor-targeted functional inhibition of Pms2 may be a valuable strategy for increasing the efficacy of anticancer agents in the treatment of p53-mutant cancers [70].

In the present study, we discovered that shMTHFR induced MLH1 in folate depletion, suggesting that reduced MTHFR function could be involved in MLH1 mediated DNA repair. In gastric cancer cell MKN45, knockdown of MTHFR in gastric cancer cell MKN45 decreased cell survival and resulted in cell cycle arrest at the G2 phase. Overexpression of MTHFR in human gastric cancer cell MKN45 was found to downregulate hMLH1 [71]. These cells also had lower levels of c-myc expression, and overexpression of MTHFR increased cell proliferation and induced the downregulation of p21WAF1 and hMLH1. These data implied that c-myc and p21WAF1 could also be involved in the better DNA stability in shMTHFR of human HCC, but further studies are certainly needed. How MTHFR function regulates the interactions among p53, MLH1, and PSM2 in the DNA repair system in human HCC will be investigated in the future.

In summary, MTHFR knockdown assists liver-origin cell defense against folate depletion-induced chromosome segregation and uracil misincorporation in the DNA by prolonging the G2/M cell cycle and promoting nuclear thymidine synthesis multi-enzyme complex formation and nuclear DNA repair proteins’ MLH1/p53 expression.

## 4. Materials and Methods

### 4.1. mRNA Expression of MTHFR and Survival Rate in HCC Patients

The expression analysis of MTHFR mRNA in HCC tumors and normal tissues was examined by the GEPIA web tool (http://gepia2.cancer-pku.cn/#index, accessed on 14 May 2021). The median of MTHFR mRNA expression level in HCC was compared to the TCGA normal data as well as to the TCGA normal and GTEx data.

When comparing gene expression between tumor and normal tissues using different datasets from public resources, one cannot rule out the possibility of systemic differences between different datasets, therefore we further compared the MTHFR gene expression in a RNA-Seq dataset containing 42 paired tumor and tumor-adjacent normal HCC tissues generated from the Cancer RNA-Seq Nexus on 14 May 2021 (CRN, http://syslab4.nchu.edu.tw/CRN) [36,37]. The mean MTHFR gene expression level (fragments per kilobase per million, FPKM) of tumor tissues were compared between the HCC tumor and the adjacent normal tissues by Student’s *t*-test.

The survival analysis was performed using the Pan-cancer RNA-Seq Web server (http://kmplot.com/analysis/index.php?p=service&cancer=liver_rnaseq) on 14 May 2021) for generating Kaplan–Meier plots by auto-selecting the best cutoff values between lower and upper quartiles into high and low expression groups that included all stages, sex, race, and mutation burden [38]. The Kaplan–Meier survival analysis was performed on the HCC RNA-seq data of the TCGA/GTEx datasets available within GEPIA2, by auto-selecting the best cutoff values into high and low expression.

### 4.2. Establishment of MTHFR Knockdown Cell-Line by RNA Interference

The MTHFR knockdown HepG2 cell lines were established using the lentiviral small hairpin RNA (shRNA) interference vectors (National RNAi Core Facility, Institute of Molecular Biology, Academia Sinica, Taipei, Taiwan). The shRNA constructs were based on the pLKO.1-puro vector and these lentivirus-based shRNA constructs. The target sequences of various shRNAs are listed in Table 1A and the target sites are provided in Supplementary Figure S1.

The different shRNA lentiviruses were produced from HEK293T packaging cells that were transfected (Lipofectin from Invitrogen, Carlsbad, CA, USA) separately with either MTHFR shRNA (sh3′UTR, sh77, sh546, sh697, sh1618) or empty EGFP lentiviral plasmids, prepared by the PureYield Plasmid kit (Promega, Madison, WI, USA). To do so, virus-producer HEK 293T cells were seeded (1.5–2.0 × 10^5^ cells per well in a 24-well plate) 1 day before transfection. Cells were transfected by using 1.5 μL of Lipofectamine 2000 (Invitrogen, Carlsbad, CA, USA) with the plasmid DNAs. The transfected GFP-expressing cells were examined under a fluorescence microscope (Supplemental Figure S2A–C), and the GFP fluorescence intensity was measured by flow cytometry (Cytomics FC 500; Beckman Coulter, Pasadena, CA, USA). The number of GFP-positive cells (IU: infectious unit) was divided by the volume of viral solution (mL) to calculate virus titer. The flow cytometry results of the empty lentiviral EGFP vector showed an estimation of 99% transfection efficiency (Supplemental Figure S2D,E), proving that a successful HEK 293T packaging line was established using this lentiviral system.

After the transfection, the supernatants of different transfected cells were collected 48 h post-transfection and centrifuged at 800× *g* for 15 min at 4 °C to remove cell debris. Ten-fold dilution of virus solutions were used to infect HepG2 cells (Bioresource Collection and Research Center, Hsinchu, Taiwan). Forty-eight hours after the virus infection, the transduced HepG2 cells underwent puromycin selection (20 μg/mL puromycin from Sigma (Ronkonkoma, NY, USA) for at least 2 weeks to generate individual stable clones. The surviving cells were cultivated and maintained for further applications.

### 4.3. Cell Culture Conditions

All chemicals were purchased from Sigma-Aldrich Chemical Company (St. Louis, MO, USA) unless otherwise specified. Human hepatoma cell line HepG2 was grown in α-Modified Eagle’s Medium or RPMI Medium with 10% bovine serum, Pen-Strep-Ampho solution [100,000 units/L Penicillin, 100 mg/L Streptomycin, 0.25 mg/mL Amphotericin]. Human embryonic kidney cell line 293T (courtesy of Dr. Shih-Lan Hsu from Taichung Veterans General Hospital) was maintained in Dulbecco’s Modified Eagle’s Medium supplemented with fetal bovine serum (FBS) (TerraCell International, ON, Canada), Pen-Strep-Ampho solution [100,000 units/L Penicillin, 100 mg/L Streptomycin, 0.25 mg/mL Amphotericin]. Cells were incubated at 37 °C in a humidified atmosphere containing 5% CO2. The media were replaced every 72 h.

The interactions between shMTHFR and folate insufficiency were investigated as follows. In the cell cycle and SAM analysis, “no folate cells” were cultured in folate depletion medium (no folate RPMI-1640, Gibco Invitrogen, Carlsbad, CA, USA) for 12 days; “folate replete” cells were cultured in folate sufficient RPMI-1640 medium (with 2.2 μM colic acid) for 12 days. In the low-folate experiments for SAM analysis, cells were cultured in folate depletion medium with 36.7 μM hypoxanthine and 37.1 μM thymidine for 9 days (to accelerate the depletion of intracellular folate content that was divided in half during cell division), then treated in medium with 2 nM folinate for 3 days (to generate a steady-state of low intracellular folate) with the same hypoxanthine and thymidine levels. In the folate depletion experiments for cytosol and nucleus protein expression, “folate deplete cells” were cultured in RPMI-1640 without folate medium for 11 days; “folate replete” cells were cultured in folate-sufficient RPMI-1640 for 11 days.

In the folate-replete/deplete stable isotope labeling experiments, control cells were cultured in folate-replete RPMI-1640 medium for 11 days. Folate-depleted cells (-FA) were cultured in RPMI-1640 without folate medium for 11 days. Folinate has been shown to be effective in rescuing certain impaired 1C metabolic pathways induced by methotrexate [45] or folate depletion [47]. The folate-replete and -deplete cells (-FA + folinate group) were cultured in no-folate medium for 8 days and then supplemented with 10 nM folinate for 3 days. These levels were based on our previous experiments and were used to generate mildly low intracellular folate [26,48].

In the folate deficiency-induced micronuclei and uracil misincorporation experiments, “folate repleted” cells were cultured in folate-sufficient medium for 12 days; “low folate” cells were cultured in folate depletion medium for 9 days and then in 2 nM folinate for 3 days; “no folate” cells were cultured in no-folate RPMI medium for 12 days, which was modified based on our previous experiment [8].

### 4.4. MTHFR Gene Expression in HepG2 Cells with shMTHFR

Five different homologous human MTHFR shRNA target sequences were used to generate different lentivirus clones termed (1) sh3′UTR, (2) sh77, (3) sh546, (4) sh697, (5) sh1618 that represented the target site on the MTHFR cDNA sequence. The shGFP clone was used as the negative control cell-line (Neg) as it underwent the same lentiviral transfection procedure but it did not target a specific human gene sequence. The relative efficiency of different target shRNA sequences on the reduction of MTHFR expression was determined by real-time PCR.

To determine the efficiency of each shRNA on MTHFR mRNA expression, total RNA form was extracted by TRIZOL reagent (Invitrogen, Carlsbad, CA, USA) and integrity was checked by electrophoresis [40]. RNA was then reversely transcribed with random primers following the manufacturer’s protocol and cDNA from each cell lines were used as templates for quantitative PCR using SYBR green gene expression assay with predesigned primers for human MTHFR. Real-time quantitative PCR was performed using Prism 7000 (Applied Biosystem Inc., Foster City, CA). The MTHFR mRNA expression was calculated by normalizing the threshold cycle value of the target gene to that of the control housekeeping gene (18sRNA). The relative MTHFR expression was given by the formula: 2^−^^ΔΔ^^CT^, where ΔΔCT = ΔC_T_RNAi clones—ΔC_T_ negative control; with ΔCT = ΔCT *_MTHFR_*—ΔCT _18S_. Relative efficiency of different target shRNA sequences on the reduction of MTHFR compared to wildtype (WT, as 100%) are shown in Table 1. Clone sh77 that had the most significant reduction (by ~63%) in MTHFR mRNA expression was used to further study the efficiency on MTHFR protein expression, MTHFR enzyme activity, and its impacts in combination with folate depletion.

### 4.5. Effects of siRNA on MTHFR Protein Expression

Effects of siRNA on MTHFR protein expression were examined by Western blot. Cell lysates were prepared [72]. Protein lysates were resolved on 10% SDS polyacrylamide gel, electro-transferred to polyvinylidene fluoride membranes, and blocked in 5% non-fat dry milk in Tris-buffered saline, pH 7.5 (100 mM NaCl, 50 mM Tris, and 0.1% Tween-20). Membranes were immunoblotted overnight at 4 °C with anti-MTHFR polyclonal antibody (Santa Cruz, CA. USA), and anti-β-actin antibody (Sigma-Aldrich, St. Louis, MO, USA). Washed blots were incubated with Immobilon Western Chemiluminescent HRP Substrate (Millipore) according to the manufacturer’s instructions, followed by the detection with a Koda fluorescence scanner. Values are expressed as mean ± SD (*n* = 3). The data were analyzed by one-way ANOVA. Different letters indicate statistical differences (*p* < 0.05) among different groups.

### 4.6. Effects of siRNA on MTHFR Enzyme Activity

Specific MTHFR activity was compared between WT, Neg, and shMTHFR cells using the ^14^C-labeled methyltetrahydrofolate (^14^C-CH_3_THF)–menadione oxidoreductase assay [73] with modifications. Cell extracts were incubated for 60 min at 37 °C in a reaction mixture containing 0.18 mol/L phosphate buffer, 3.5 mmol/L menadione, 1.4 mmol/L EDTA, 7.6 mmol/L ascorbic acid, 70 µmol/L FAD, and 300 µmol/L [^14^C]CH_3_THF in a total volume of 143 µL. The reaction was terminated by the addition of 125 µL 0.6 mol/L sodium acetate, pH 4.5. After the addition of 50 µL 100 mmol/L formaldehyde and 75 µL 0.4 mol/L dimedone, the mixture was boiled for 12 min and subsequently cooled on ice. For each sample, 2.5 mL toluene was added and the tubes were vigorously vortexed twice for 15 s. After centrifugation, the formation of the radio-labeled [^14^C] formaldehyde-dimedone adduction was quantified by scintillation counting of the supernatant. Enzyme activity was expressed as nanomoles of formaldehyde formed per hour per milligram of protein.

### 4.7. Cell Cycle Analyses

A total of 5 × 10^6^ cells were WT, Neg, and shMTHFR HepG2 cells harvested, washed, and resuspended in cold PBS with ice-cold ethanol, and then washed with Ca2+/Mg2+ free HBSS containing 1% BSA [74]. The cells were then incubated in 50 mg/mL propidium iodide (PI; Sigma, St. Louis, MO, USA) containing 1 mg/mL sodium citrate, 100 mg/mL RNase I and 0.1% Triton X-100 for 30 min at 37 °C, and analyzed by flow cytometry in fluorescence-activated cell sorter (Epics XL.MCL, Beckman Coulter, Inc, Fullerton, CA, USA) using the EXPO32 software.

### 4.8. Determination of SAM and SAH Contents

The impacts of folate restriction on SAM were examined separately in each cell line and compared to the same cell line under folate-replete conditions. After the incubation period, cells were harvested, washed, and pelleted by centrifugation. Cell extracts for SAM and SAH analysis were prepared by a modification of the previously described procedure. Cells were centrifuged and washed with cold PBS twice while being kept on ice. PBS was carefully aspirated and cell pellets were resuspended in 0.4 M ice-cold perchloric acid. Pellets were hand-homogenized on ice with a hand-held mini pestle [26]. Homogenates were centrifuged at 4 °C and supernatants were collected and stored at −80 °C for analysis by HPLC as described by Fell [75]. The supernatant of each sample was filtered through 0.45 μ M and then loaded onto a C18 column (250 × 4.6 mm), fitted with a matched guard column operated by a Hitachi L-7100 intelligent pump connected to an L-7400 UV detector. The absorption of eluted compounds was monitored using ex = 254 nm [40].

SAM and SAH values were normalized to total cell numbers [5]. In folate depletion experiments, cells were grown without folate-developed macrocytosis, so the calculation was normalized to protein contents instead of cell number [8]. Cellular protein contents were determined by the bicinchoninic assay (Pierce, Rockford, IL, USA).

### 4.9. Western Blot Analyses

After cells were harvested, nuclear and cytosol proteins were isolated and fractionated using the nuclear/cytosol fractionation Kit (Nuclear/Cytosol Fractionation Kit, Catalog #K266, BioVision, Milpitas, CA, USA, San Francisco) following the manufacturer’s instructions [76]. Protein was quantified using a BCA assay kit (Pierce, Rockford, IL, USA, Winnebago). For Western blots, proteins were separated by SDS-PAGE gel, transferred to polyvinylidene difluoride membrane, and blotted with a designated antibody according to the manufacturer’s instructions. Equal loading and/or purity of nuclear fractions was confirmed through the detection of Lamin A using α-Lamin A (GeneTex, Irvine, CA, USA, Orange, 1∶1000 dilution) [77], and tubulin using α-tubulin (Abcam, Cambridge, non-metropolitan county, UK, Cambridgeshire, 1∶5000 dilution). No cross-contamination between the nuclear and cytoplasmic fractions was observed. Thirty micrograms of proteins (from folate depletion experiments) or 16 µg of proteins (from folate repletion experiments) were separated by SDS-PAGE (12% gel) and subsequently transferred to a polyvinylidene difluoride membrane and blotted with primary antibodies, including anti-Thymidylate synthase (1:1000; sc-376161; Santa Cruz, Dallas, TX, USA), anti-DHFR (1:1000; ab133546; Abcam, Cambridge, non-metropolitan county, UK, Cambridgeshire), anti-a-Tubulin (1:5000; NB100-690; Novus, Littleton, CO, USA), anti-MTHFD1 (1:500; sc-134732; Santa Cruz, Dallas, TX, USA), anti-MLH1 (1:1000; sc-271978; Santa Cruz, Dallas, TX, USA), anti-SHMT1 (1:1000; #80715; Cell Signaling, Danvers, MA, USA, Essex), anti-Actin (1:5000; NB600-501; Novus, Littleton, CO, USA), anti–Lamin A/C (1:1000; GTX101127; GeneTex, Irvine, CA, USA, Orange), anti-p53 (1:1000; #9282; Cell Signaling, Danvers, MA, USA, Essex), followed by incubation with HRP-conjugated polyclonal secondary antibody (1:2000; ab6721; Abcam, Cambridge, non-metropolitan county, UK). All Western blots were visualized using the enhanced plus chemiluminescence assay kit (Adv, San Jose, CA, USA, San Francisco), according to the manufacturer’s protocol. Protein expression levels were quantified by ImageJ software (Analytik Jena US LLC, Upland, CA, USA, San Bernardino).

### 4.10. Stable Isotope Labeling Experiments

To investigate the impacts of shMTHFR on purine and thymidylate synthesis under different folates conditions, kinetic experiments were conducted based on our previous studies [5,26,45] with modifications for the present study. Stable isotopic tracer [3-^13^C]-serine (Cambridge Isotope Laboratories, Woburn, MA, USA) was used to trace the 1-carbon flow in the subsequent experiments as the β-carbon of serine provides the major 1-carbon source in folate metabolic pathways.

After the treatment period, cells were harvested and total genomic DNA was extracted using a standard phenol/chloroform/isoamyl alcohol procedure with RNase treatment to remove RNA. DNA was dried under nitrogen and hydrolyzed in formic acid under vacuum. The dried DNA samples were then converted into trimethylsilane-base derivatives which were then separated on an HP-5MS column. Isotopic enrichments in the nucleotides were determined in positive ionization mode for nucleotides and negative ionization mode for amino acids by GC/MS using a model 6890 gas chromatography and model 5973 mass spectrometer (Agilent, Palo Alto, CA, USA). Selected ion monitoring was conducted at a *m/z* 255–257 for thymine, *m/z* 280–283 for adenine, and *m/z* 368–371 for guanine.

### 4.11. Determination of Micronuclei

After the designated treatment period, cells were fixed with 70% ethanol and then stained using EtBr and visualized under a digital fluorescence microscope for the identification of MNi. Nuclei were considered as MNi according to established criteria including the following: (1) diameter of the MN less than one third of the main nucleus; (2) MNi was separated from the main nuclei with distinguishable nuclear boundary, and (3) MNi exhibited similar staining intensity as the main nucleus [1].

MN frequency was obtained by counting the number of MN-positive cells among 500 total cells in each treatment group, and finally, the percentage of MN was calculated from the data obtained.

### 4.12. Determination of Uracil Content in the DNA

Genomic DNA was extracted using a standard phenol/chloroform/isoamyl alcohol procedure with RNase treatment to remove RNA. DNA samples were then incubated with uracil DNA glycosylase (New England Biolab, Ipswich, MA, USA). An internal standard of [15N2]uracil (kindly provided by Professor Stover at Texas A&M University) was added before the uracil was extracted and derivatized. Analysis of uracil-3,5-bis(trifluoromethyl)benzyl bromide was carried out by 6890-GC coupled with 5975C-MS17, 39 (Agilent Technology, Palo Alto, CA, USA). Separation of derived uracil was achieved on an HP-5MS (30 m, 0.25 mm) column. Ionization was achieved using the NCI mode and monitoring at *m/z* 337 for uracil and 339 for [15N2]uracil. The amount of uracil in DNA was presented as pg uracil/µg DNA [8]

### 4.13. Statistical Analysis

For cell culture data analyses, the differences amongst cells with different genotypes were examined by one-way ANOVA, then the comparisons of means between each of the two groups were determined using post hoc analyses. All statistical analyses were performed with SYSTAT 11.0 for Windows^TM^ (Systat Software Inc., Richmond, CA, USA). For all analyses, the results were considered statistically significant if *p*-values were <0.05.

## 5. Conclusions

In conclusion, MTHFR knockdown assists HCC cell defense against folate depletion-induced chromosome segregation and uracil misincorporation in the DNA by prolonging the G2/M cell cycle and promoting nuclear thymidine synthesis multi-enzyme complex formation and nuclear DNA repair proteins’ MLH1/p53 expression.

## Figures and Tables

**Table 1:** MTHFR knockdown alters cell cycle distribution (%) in HepG2 cells.

(**A**)							
shRNA (Clone ID)		Target sequence				Source	
TRCN0000046468/sh3′UTR		CCTCAGTTTCTCCATCAGCTT				National RNAi Core Facility, Academia Sinica, Taiwan	
TRCN0000046469/sh697		GCTGACACATTCTTCCGCTTT					
TRCN0000046470/sh77		CCAAAGATAGTTCGAGATGTT					
TRCN0000046471/sh546		CCGAAGTGAGTTTGGTGACTA					
TRCN0000046472/sh1618		CTTGTCAATGTGAAGGGTGAA					
TRCN0000072178/shGFP/Neg		CAACAGCCACAACGTCTATAT					
(**B**) Gene expression and cell cycle distribution							
Cell-line	MTHFR expression	G1 (%) ^1^		S (%) ^1^		G2/M (%) ^1^	
Wild type	100 %	71.2 ± 0.6 ^ab^		19.7 ± 1.0 ^f^		9.1 ± 0.6 ^m^	
RNAi shGFP ^1^	110 ± 1%	73.8 ± 2.3 ^a^		17.3± 1.9 ^fgh^		8.9 ± 0.5 ^m^	
RNAi sh3′UTR ^1^	96 ± 10%	70.1 ± 1.1 ^bc^		14.0 ± 0.2 ^i^		15.9 ± 1.0 ^k^	
RNAi sh1618 ^1^	61 ± 2%	68.5 ± 1.0 ^bc^		17.8 ± 0.6 ^gh^		13.5 ± 1.1 ^l^	
RNAi sh546 ^1^	44 ± 3%	64.8 ± 1.0 ^de^		18.0 ± 1.7 ^fg^		17.1 ± 0.3 ^k^	
RNAi sh697 ^1^	42 ± 1%	67.3 ± 0.7 ^cd^		16.1 ± 0.2 ^ghi^		16.6 ± 0.8 ^k^	
RNAi sh77 ^1^	37 ± 6%	63.5 ± 3.6 ^e^		14.9 ± 3.2 ^hi^		21.6 ± 0.4 ^j^	
(**C**) The associations between MTHFR gene expression and cell cycle distributions ^2^							
		G1 (%)		S (%)		G2/M (%)	
	n	r^2^	*p*-value ^2^	r^2^	*p*-value ^2^	r^2^	*p*-value ^2^
MTHFR mRNA	7	0.86	<0.0001	0.034	0.907	−0.808	<0.0001
(**D**) The associations between MTHFR gene expression and the cell cycle distribution ^2^							
MTHFR	n	G1 (%)		S (%)		G2/M (%)	
		r^2^	*p*-value ^2^	r^2^	*p*-value ^2^	r^2^	*p*-value ^2^
Gene expression	7	0.86	<0.0001	0.03	0.91	−0.810	<0.0001
Protein expression	3	0.56	0.12	0.760	0.020	−0.800	0.010
Enzyme Activity	3	0.592	0.094	0.812	0.008	−0.842	0.005

^1^ Values are expressed as mean ± SD for triplicate samples. Different letters in the same column indicate statistically differences (*p* < 0.05) among different groups. ^2^ Correlations were determined by *Pearson’s* correlation, r: correlation coefficient; *p*-value: probabilities. Different letters in the same column indicate statistically differences (*p* < 0.05) among different groups (*p* < 0.05).

**Table 2:** MTHFR knockdown alters intracellular SAM and SAH contents in HepG2 cells. (A) MTHFR knockdown significantly decreased intracellular SAM contents and SAM to SAH ratio; (B) the associations between MTHFR mRNA, protein, enzyme activity and SAM, SAH, Hcy and cysteine concentrations in HepG2 cells.

(**A**) ^2^	SAM ^1^		%	SAH ^1^	%	SAM: SAH ratio ^1^ %	
Wt ^1^	438.5 ± 18.0 ^a^		−30.8% ^3^	16.39 ± 0.46	NS	26.75 ^d^	−20.0% ^3,^*
Neg ^1^	523.1 ± 59.4 ^a^		−42.0% ^4^	15.98 ± 0.36	NS	32.72 ^e^	−34.6% ^4^
shMTHFR ^1^	303.5 ± 75.4 ^b^			14.18 ± 2.86		21.41 ^d^	
(**B**)		SAM		SAH		SAM: SAH ratio ^1^	
MTHFR ^1^	n	r ^7^	*p*-value ^7^	R ^7^	*p*-value ^7^	r ^7^	*p*-value ^7^
mRNA ^5^	3	0.693	0.127	0.133	0.802	0.719	0.107
Protein ^6^	3	0.353	0.351	−0.153	0.694	0.362	0.339
Activity ^6^	3	0.868	0.003	0.062	0.874	0.687	0.041

^1^ Abbreviations: SAM: *S*-adenosyl methionine; SAH: *S*-adenosyl homocysteine. MTHFR: methylenetetrahydrofolate reductase; Wt: wildtype HepG2 cells, shMTHFR: clone sh77 that had ~63% in *MTHFR* mRNA expression, Neg: the negative control cell-line. ^2^ Data in (A) are expressed as mean ± SD (pmol/million cells, *n* = 3) and were analyzed by one-way ANOVA. Different letters in the same column indicate statistically differences (*p* < 0.05) among different groups. ^3^ % change of shMTHFR compared to Wt. NS: shMTHFR not significant differed from WT or Neg * A trend of reduction in shMTHFR compared to Wt (*p* = 0.07). ^4^ % change of shMTHFR compared to Neg. ^5^ n: number of observations in duplicate each. ^6^ n: number of observations in triplicate each. ^7^ r: correlation coefficient; *p*-value: probabilities.

**Table 3:** Effects of MTHFR knockdown and folate depletion on cell cycle distribution and SAM, SAH contents in HepG2 cells.

(**A**) Cell cycle distributions in folate deficiency in Wt, Neg, and shMTHFR cells				
Genotype		G1 (%)	S (%)	G2/M (%)
	Folate replete ^2^	71.2 ± 1.1 ^a^	20.4 ± 1.1 ^ef^	9.1 ± 1.2 ^k^
Wt ^1^	No folate ^2^	50.3 ± 4.2 ^c^	37.2 ± 4.2 ^g^	14.2 ± 1.3 ^j^
	% change	−21 ± 4	+17 ± 4	+4 ± 1
	Folate replete ^2^	74. ± 2 ^a^	18.2 ± 2.1 ^ef^	9.1 ± 1.1 ^k^
Neg ^1^	No folate ^2^	67.6 ± 2 ^ab^	20.6 ± 1.4 ^e^	13.6 ± 1 ^j^
	% change	−6 ± 2	3 ± 11	+4 ± 1
	Folate replete ^2^	64.3 ± 4 ^b^	15.7 ± 3.1 ^f^	22.2 ± 0.4 ^h^
shMTHFR ^1^	No folate ^2^	49.4 ± 3 ^c^	33.2 ± 4.2 ^g^	19.5 ± 1.3 ^i^
	% change	−15 ± 3	+18.1 ± 4	−3 ± 1
(**B**) SAM and SAH contents in folate deficiency in Wt, Neg, and shMTHFR cells				
Genotype		SAM ^4^	SAH ^4^	SAM/SAH ^5^
	Folate replete	2457.3 ± 237.2 ^a^	92.3 ± 14.1 ^a^	26.8 ± 1.6 ^e^
Wt ^1^	Low Folate ^3^	1753.6 ± 289.0 ^b^	78.1 ± 1.7 ^a^	22.4 ± 3.6 ^ef^
	No folate	19,44.1 ± 501.8 ^ab^	406.2 ± 101.4 ^b^	4.78 ± 0.04 ^g^
	Folate replete	2577.9 ± 67.7 ^a^	79.3 ± 7.4 ^a^	32.7 ± 3.03 ^h^
Neg ^1^	Low Folate ^3^	1444.0 ± 257.0 ^b^	72.1 ± 16.0 ^a^	20.2 ± 0.9 ^ef^
	No folate	2076.8 ± 623.6 ^ab^	109.1 ± 2.6 ^a^	19.1 ± 6.1 ^ef^
	Folate replete	2021.9 ± 546.6 ^ab^	97.3 ± 34.6 ^a^	21.5 ± 3.55 ^f^
shMTHFR ^1^	Low Folate ^3^	1618.5 ± 267.1 ^b^	120.8 ± 46.5 ^a^	14.1 ± 2.9 ^i^
	No folate	1696.2 ± 277.0 ^b^	108.9 ± 5.4 ^a^	15.6 ± 2.7 ^i^

^1^ Cell-lines: Transduction with (HepG2 shMTHFR) or without (HepG2WT) MTHFR RNAi virus clones and a negative control cell line (Neg) were studied. ^2^ Folate replete: Treated in folate sufficient medium for 12 days; No folate: in folate depletion medium for 12 days. ^3^ Low folate condition: cells were cultured in folate depletion medium with 36.7 μM hypoxanthine and 37.1 μM thymidine for 9 days, then treated in medium with 2 nM folinate with hypoxanthine and thymidine for 3 days. ^4^ SAM: *S*-adenosyl methionine. SAH: *S*-adenosyl homocysteine. Data are expressed as (pmol/protein mg). Cells grown without folate developed macrocytosis, so the calculation was normalized to protein contents instead of cell number in the folate depletion experiments. ^5^ SAM/SAH: The ratio of S-adenosyl methionine to S-adenosyl homocysteine. ^6^ Values are expressed as mean ± SD (*n =* 3). The data were analyzed by one-way ANOVA. Different letters in the same column indicate statistically differences (*p* < 0.05) among different groups.

**Table 4:** shMTHFR enhanced cytosolic and nuclear SHMT/DHFR/TYMS ^1^ protein expression ^2^ in HepG2 cells.

(**A**) Cytosol				
Cytosol				
Folate replete ^4^	SHMT1	DHFR	TYMS	MTHFD
WT ^3^	0.329 ± 0.038 ^a^	0.237 ± 0.032 ^ae^	0.293 ± 0.000 ^a^	0.340 ± 0.011 ^a^
Neg ^3^	0.197 ± 0.197 ^c^	0.306 ± 0.016 ^a^	0.204 ± 0.054 ^abce^	0.227 ± 0.011 ^c^
shMTHFR ^3^	0.411 ± 0.411 ^abf^*	0.289 ± 0.000 ^a^	0.320 ± 0.006 ^bc^*	0.370 ± 0.011 ^ab^*
Cytosol				
Folate deplete ^4^	SHMT1	DHFR	TYMS	MTHFD
WT ^3^	0.081 ± 0.029 ^d^	0.170 ± 0.029 ^de^	0.186 ± 0.013 ^d^	0.135 ± 0.022 ^d^
Neg ^3^	0.064 ± 0.001 ^df^	0.089 ± 0.016 ^df^	0.106 ± 0.015 ^f^	0.162 ± 0.023 ^def^
shMTHFR ^3^	0.355 ± 0.029 ^ae^	0.241 ± 0.022 ^e^*	0.208 ± 0.001 ^de^*	0.203 ± 0.000 ^e^
(**B**) Nucleus				
Nucleus				
Folate replete ^4^	SHMT1	DHFR	TYMS	MTHFD1
WT ^3^	0.137 ± 0.020 ^a^	0.024 ± 0.000 ^a^	0.086 ± 0.020 ^a^	0.321 ± 0.001 ^a^
Neg ^3^	0.105 ± 0.004 ^a^	0.019 ± 0.001 ^ac^	0.074 ± 0.028 ^a^	0.216 ± 0.001 ^c^
shMTHFR ^3^	0.123 ± 0.006 ^a^	0.064 ± 0.001 ^b^	*0.100 ± 0.007 ^a^*	0.400 ± 0.006 ^b^
Nucleus				
Folate deplete ^4^	SHMT1	DHFR	TYMS	MTHFD1
WT ^3^	0.162 ± 0.033 ^de^	0.159 ± 0.019 ^de^	0.115 ± 0.071 ^de^	0.141 ± 0.003 ^df^
Neg ^3^	0.093 ± 0.019 ^df^	0.096 ± 0.024 ^df^	0.096 ± 0.040 ^df^	0.129 ± 0.040 ^ef^
shMTHFR ^3^	0.245 ± 0.044 ^e^*	0.206 ± 0.000 ^e^*	0.289 ± 0.022 ^e^*	0.231 ± 0.013 ^e^

^1^ SHMT: serine hydroxymethyltransferase; DHFR: dihydrofolate reductase; TYMS: thymidylate synthase. ^2.^ Protein expressions were determined by Western blot. Values are expressed as mean ± SD (*n* = 2–3). The data were analyzed by one-way ANOVA. Different letters in the same column indicate statistically differences (*p* < 0.05) among different groups. Bold values indicate that shMTHFR is statistically different from Wt or Neg (*p* < 0.05). * indicated a trend of difference (*p* < 0.1) compared to Wt or Neg. ^3.^ WT: wildtype HepG2 cells; Neg: negative control cells; shMTHFR: HepG2 cells with MTHFR knockdown by RNA interference. ^4^ Folate replete: cultured in folate replete RPMI-1640 medium for 11 days; folate deplete: cultured in folate depletion medium (RPMI-1640 with no folate media) for 11 days.

**Table 5:** MTHFR knockdown assisted nucleotide biosynthesis in HepG2 cells ^1,2^.

	dA + 1 ^4^	dA + 2 ^4^	dA (MIA) ^4^	dG + 1 ^4^	dG + 2 ^4^	dG (MIA) ^4^	dT + 1 ^5^
Wt ^2^							
Control ^3^	0.275 ± 0.000 ^a^	0.056 ± 0.000 ^ab^	0.413 ± 0.005 ^ab^	0.287 ± 0.000 ^a^	0.052 ± 0.000 ^a^	0.362 ± 0.000 ^a^	0.244 ± 0.001 ^a^
−FA ^3^	0.173 ± 0.007 ^d^	0.025 ± 0.000 ^d^	0.294 ± 0.003 ^df^	0.188 ± 0.007 ^d^	0.022 ± 0.00 ^d^	0.240 ± 0.005 ^d^	0.217 ± 0.005 ^d^
−FA + folinate ^3^	0.348 ± 0.001 ^gh^	0.086 ± 0.000 ^g^	0.499 ± 0.000 ^g^	0.340 ± 0.005 ^ghi^	0.074 ± 0.004 ^g^	0.439 ± 0.018 ^g^	0.278 ± 0.002 ^gi^
Neg ^2^							
Control ^3^	0.291 ± 0.001 ^bc^	0.066 ± 0.001 ^c^	0.455 ± 0.005 ^c^	0.290 ± 0.006 ^a^	0.056 ± 0.003 ^a^	0.389 ± 0.035 ^ac^	0.270 ± 0.002 ^c^
−FA ^3^	0.135 ± 0.003 ^f^	0.020 ± 0.001 ^f^	0.302 ± 0.011 ^f^	0.154 ± 0.004 ^f^	0.018 ± 0.002 ^f^	0.239 ± 0.026 ^df^	0.177 ± 0.001 ^f^
−FA + folinate ^3^	0.343 ± 0.002 ^gi^	0.089 ± 0.000 ^i^	0.522 ± 0.001 ^i^	0.328 ± 0.002 ^i^	0.077 ± 0.003 ^g^	0.470 ± 0.015 ^gi^	0.284 ± 0.001 ^i^
shMTHFR ^2^							
Control ^3^	0.269 ± 0.007 ^ab^	0.059 ± 0.003 ^bc^	0.442 ± 0.016 ^ac^	0.284 ± 0.006 ^a^	0.059 ± 0.002 ^a^	0.419 ± 0.011 ^b^	0.255 ± 0.001 ^b^
−FA ^3^	0.179 ± 0.001 ^de^	0.032 ± 0.000 ^e^	0.358 ± 0.008 ^e^	0.197 ± 0.003 ^de^	0.029 ± 0.000 ^e^	0.298 ± 0.000 ^e^	0.216 ± 0.001 ^de^
−FA + folinate ^3^	0.355 ± 0.002 ^h^	0.097 ± 0.000 ^h^	0.550 ± 0.003 ^h^	0.347 ± 0.005 ^h^	0.087 ± 0.000 ^g^	0.501 ± 0.008 ^h^	0.292 ± 0.000 ^h^

^1^ Values are enrichments from [3-^13^C]-serine labeling experiments. Data are presented as mean ± SD (*n* =2~3/group). Different letters in the same column indicate statistically differences by one-way ANOVA (*p* < 0.05). ^2^ WT: wildtype HepG2 cells; Neg: negative control cells; shMTHFR: HepG2 cells with MTHFR knockdown by RNA interference. dA: deoxyadenosine enrichments; dG: deoxyguanosine; dT: deoxythymidine. ^3^ +FA: cells were cultured in folate replete RPMI-1640 medium for 11 days; −FA: cells were cultured in folate depletion medium (RPMI-1640 with no folate media) for 11 days; −FA + folinate: cells were cultured in folate depletion medium for 8 days and then supplement with folinate for 3 days. ^4^ Incorporation of serine derived formate into either C2 or C8 purine ring appeared as M + 1 specie; incorporation of serine derived formate into both C2 and C8 purine ring appeared as M + 2 specie of purine. MIA: determined from the ratio of the M + 1 and M + 2 isomers of dA and dG. A value of 1.0 would indicate that 100% of the C2 and C8 carbons of the purine ring were derived from [3-^13^C]-serine derived formate. ^5^ Incorporation of one carbon moiety into deoxythymidine from [3-^13^C]-serine.

**Table 6:** shMTHFR reduced folate deficiency induced micronuclei formation and DNA uracil misincorporation in HepG2 cells.

(**A**) Micronuclei determined by cytokinesis-blocked micronucleus (CBMN) assay				
Number of micronuclei (MNi)/500 counts ^1^				
Cell line ^2^	Folate replete ^3^	Low folate ^3^	No folate ^3^	
WT	16.3 ± 3.1 ^a^	56.7 ± 2.5 ^d^	106.7 ± 9.0 ^g^	
Neg	25.0 ± 4.0 ^c^	71.0 ± 1.0 ^f^	68.0 ± 1.0 ^h^	
shMTHFR	6.0 ± 3.6 ^b^	27.3 ± 2.3 ^e^	28.3 ± 10.0 ^i^	
(**B**) Uracil contents in the DNA ^4^				
	Folate replete ^3^	No folate ^3^	*p*-value ^5^	% change ^5^
WT	1.34 ± 0.02	2.14 ± 0.02	<0.001	+60.1 ± 1.69
Neg	1.33 ± 0.02	2.16 ± 0.01	<0.001	+62.4 ± 1.12
shMTHFR	1.24 ± 0.02	1.79 ± 0.08	<0.001	+45.1 ± 6.56
Neg vs. WT				
*p*-value	0.642	0.304		
% change	−0.50 ± 1.35	0.87 ± 0.69		
shMTHFR vs. WT				
*p*-value	0.002	0.002		
% change	−7.44 ± 1.20	−16.2 ± 3.79		
shMTHFR vs. Neg				
*p*-value	0.002	0.001		
% change	−6.97 ± 1.21	−16.9 ± 3.76		
2-Way ANOVA ^6^				
MTHFR effect			<0.001	
Folate effect			<0.001	
MTHFR x Folate			<0.001	
(**C**) shMTHFR induced p53, MLH1 proteins expression in cytosol and nuclear ^7^				
	Cytosol		Nuclear	
Folate replete ^8^	p53	MLH1	p53	MLH1
WT	0.108 ± 0.015 ^a^	0.179 ± 0.035 ^ab^	0.126 ± 0.030 ^a^	0.278 ± 0.029 ^a^
Neg	0.239 ± 0.001 ^c^	0.173 ± 0.022 ^a^	0.218 ± 0.030 ^a^	0.248 ± 0.051 ^a^
shMTHFR	0.278 ± 0.032 ^bc#^	0.273 ± 0.007 ^b^*	0.281 ± 0.022 ^b^	0.411 ± 0.038 ^a^*^#^
	Cytosol		Nuclear	
Folate deplete ^8^	p53	MLH1	p53	MLH1
WT	0.079 ± 0.031 ^de^	0.161 ± 0.038 ^d^	0.079 ± 0.002 ^df^	0.174 ± 0.036 ^d^
Neg	0.179 ± 0.008 ^f^	0.142 ± 0.016 ^d^	0.179 ± 0.068 ^ef^	0.096 ± 0.026 ^d^
shMTHFR	0.242 ± 0.053 ^ef^*	0.197 ± 0.011 ^d^*^#^	0.242 ± 0.016 ^e^*	0.229 ± 0.001 ^e^

^1^ Values are expressed as mean ± SD for triplicate samples. Different letters in the same column indicate statistical differences (*p* < 0.05) among different groups calculated by one-way ANOVA. ^2^ Cell-lines. shMTHFR: HepG2 transduction with shMTHFR; WT: wildtype HepG2; Neg: negative control cell-line. ^3^ Culture conditions. Folate replete: cultured in folate Western blot sufficient RPMI medium for 12 days; low folate: cultured in folate depletion medium for 9 days and then in 2 nM folinate for 3 days; no folate: cultured in folate depletion medium for 12 days. ^4^ Data are expressed as mean ± SD with the unit of pg uracil/ug DNA (*n* = 3). ^5^ The *p*-value and percent changes were calculated comparing between no folate and folate replete. ^6.^ANOVA with Tukey’s post hoc test. Means without a common letter differ, *p* < 0.05. ^7^ Protein expression determined by Western blot. Values are presented as mean ± SD (*n* = 2–3/group). Different letters indicate statistical differences (*p* < 0.05) in the same column among different groups. * indicates a trend of difference between shMTHFR and WT; ^#^ indicated a trend of difference between shMTHFR and Neg (*p* < 0.1). ^8^ Folate replete: cultured in folate replete medium for 11 days; no folate: cultured in folate depletion medium for 11 days.

## Data Availability

The data presented in this study are available on request from the corresponding author.

## Supplementary Materials

The following are available online at https://www.mdpi.com/article/10.3390/ijms22179392/s1.
