# Supplementary material for: MTHFR Knockdown Assists Cell Defense against Folate Depletion Induced Chromosome Segregation and Uracil Misincorporation in DNA
Source: Int J Mol Sci. 2021 Aug 30;22(17):9392. doi: 10.3390/ijms22179392 (PMC8431311; doi:10.3390/ijms22179392)

Supplemental Figure 1.

NM_005957.3:185-2155_MTHFR_CDS

https://www.ncbi.nlm.nih.gov/nuccore/NM_005957.3?report=fasta&from=185&to=2155

ATGGTGAACGAAGCCAGAGGAAACAGCAGCCTCAACCCCTGCTTGGAGGGCAGTGCCAGCAGTGGCAGTGAGA
GCTCCAAAGATAGTTCGAGATGTTCCACCCCGGGCCTGGACCCCGAGCGGCATGAGAGACTCCGGGAGAAGAT
GAGGCGGCGATTGGAATCTGGTGACAAGTGGTTCTCCCTGGAATTCTTCCCTCCTCGAACTGCTGAGGGAGCTG
TCAATCTCATCTCAAGGTTTGACCGGATGGCAGCAGGTGGCCCCCTCTACATAGACGTGACCTGGCACCCAGCA
GGTGACCCTGGCTCAGACAAGGAGACCTCCTCCATGATGATCGCCAGCACCGCCGTGAACTACTGTGGCCTGGA
GACCATCCTGCACATGACCTGCTGCCGTCAGCGCCTGGAGGAGATCACGGGCCATCTGCACAAAGCTAAGCAG
CTGGGCCTGAAGAACATCATGGCGCTGCGGGGAGACCCAATAGGTGACCAGTGGGAAGAGGAGGAGGGAGGC
TTCAACTACGCAGTGGACCTGGTGAAGCACATCCGAAGTGAGTTTGGTGACTACTTTGACATCTGTGTGGCAGGT
TACCCCAAAGGCCACCCCGAAGCAGGGAGCTTTGAGGCTGACCTGAAGCACTTGAAGGAGAAGGTGTCTGCGG
GAGCCGATTTCATCATCACGCAGCTTTTCTTTGAGGCTGACACATTCTTCCGCTTTGTGAAGGCATGCACCGACA
TGGGCATCACTTGCCCCATCGTCCCCGGGATCTTTCCCATCCAGGGCTACCACTCCCTTCGGCAGCTTGTGAAG
CTGTCCAAGCTGGAGGTGCCACAGGAGATCAAGGACGTGATTGAGCCAATCAAAGACAACGATGCTGCCATCCG
CAACTATGGCATCGAGCTGGCCGTGAGCCTGTGCCAGGAGCTTCTGGCCAGTGGCTTGGTGCCAGGCCTCCAC
TTCTACACCCTCAACCGCGAGATGGCTACCACAGAGGTGCTGAAGCGCCTGGGGATGTGGACTGAGGACCCCA
GGCGTCCCCTACCCTGGGCTCTCAGCGCCCACCCCAAGCGCCGAGAGGAAGATGTACGTCCCATCTTCTGGGC
CTCCAGACCAAAGAGTTACATCTACCGTACCCAGGAGTGGGACGAGTTCCCTAACGGCCGCTGGGGCAATTCCT
CTTCCCCTGCCTTTGGGGAGCTGAAGGACTACTACCTCTTCTACCTGAAGAGCAAGTCCCCCAAGGAGGAGCTG
CTGAAGATGTGGGGGGAGGAGCTGACCAGTGAAGAAAGTGTCTTTGAAGTCTTCGTTCTTTACCTCTCGGGAGA
ACCAAACCGGAATGGTCACAAAGTGACTTGCCTGCCCTGGAACGATGAGCCCCTGGCGGCTGAGACCAGCCTG
CTGAAGGAGGAGCTGCTGCGGGTGAACCGCCAGGGCATCCTCACCATCAACTCACAGCCCAACATCAACGGGA
AGCCGTCCTCCGACCCCATCGTGGGCTGGGGCCCCAGCGGGGGCTATGTCTTCCAGAAGGCCTACTTAGAGTT
TTTCACTTCCCGCGAGACAGCGGAAGCACTTCTGCAAGTGCTGAAGAAGTACGAGCTCCGGGTTAATTACCACCT
TGTCAATGTGAAGGGTGAAAACATCACCAATGCCCCTGAACTGCAGCCGAATGCTGTCACTTGGGGCATCTTCCC
TGGGCGAGAGATCATCCAGCCCACCGTAGTGGATCCCGTCAGCTTCATGTTCTGGAAGGACGAGGCCTTTGCCC
TGTGGATTGAGCGGTGGGGAAAGCTGTATGAGGAGGAGTCCCCGTCCCGCACCATCATCCAGTACATCCACGAC
AACTACTTCCTGGTCAACCTGGTGGACAATGACTTCCCACTGGACAACTGCCTCTGGCAGGTGGTGGAAGACAC
ATTGGAGCTTCTCAACAGGCCCACCCAGAATGCGAGAGAAACGGAGGCTCCATGA

RNAi core clone ID	shRNA name
TRCN0000046468 (NM_005957.3:5876-5896)	sh3'UTR
TRCN0000046469	sh697
TRCN0000046470	sh77/shMTHFR
TRCN0000046471	sh546
TRCN0000046472	sh1618

Supplemental Figure 1. Target site information of shRNA on MTHFR gene.
The lentiviral RNAi system was provided by the National RNAi Core Facility, Academia Sinica, Taipei, Taiwan (http://rnai.genmed.sinica.edu.tw/). These RNAi lentivirus clones are termed (1) sh3'UTR, (2) sh77, (3) sh546, (4) sh697, (5) sh1618 that represented the target site on the MTHFR CDS sequence. The colours represent the different target sites on the MTHFR based on the version of NM_005957.3 reference sequence.

Supplemental Figure 2.

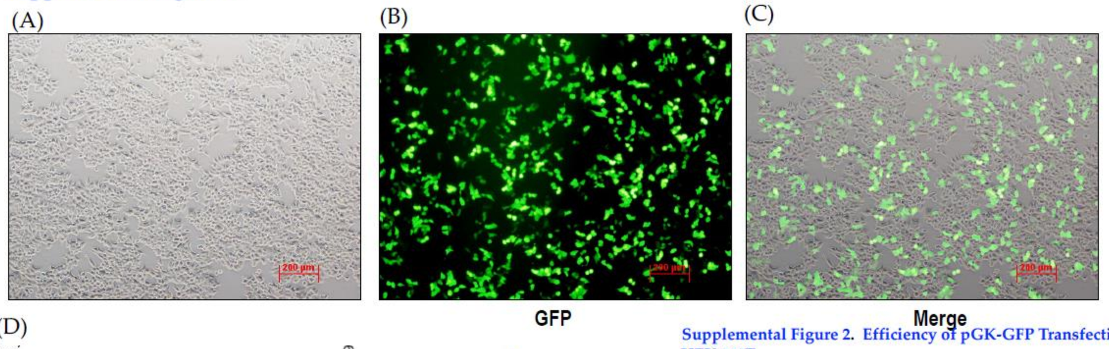

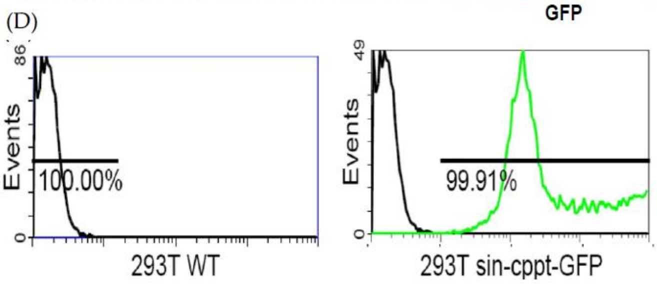

Supplemental Figure 2. Efficiency of pGK-GFP Transfection in HEK 293T.

Analysis of cells transfected with lentiviral EGFP vector for the estimation of RNAi vector transfection efficiency by fluorescence microscopic and flow cytometry. HEK 293T cells were transfected with 0.5 ug of pGK-GFP, which was derived from pLKO.1-puro. In figure (A), cells were photographed with a phase-contrast microscope. Fluorescence micrographs in figure (B) were taken using filters appropriate for EGFP. Image overlay of figure (C) indicates the presence of cells expressing EFGP. Figure (D) shows the efficiency analysis of transfection by flow cytometry. Left: GFP fluorescent analysis of 293T cells without transfected vector. Right: flow cytometry analysis of transfection efficiency, expressed by percent area excluding EGFP free HepG2 sample with transfected GFP vector.

Supplemental Figure 3 cont. Original flow cytometry data for selected Table 1B

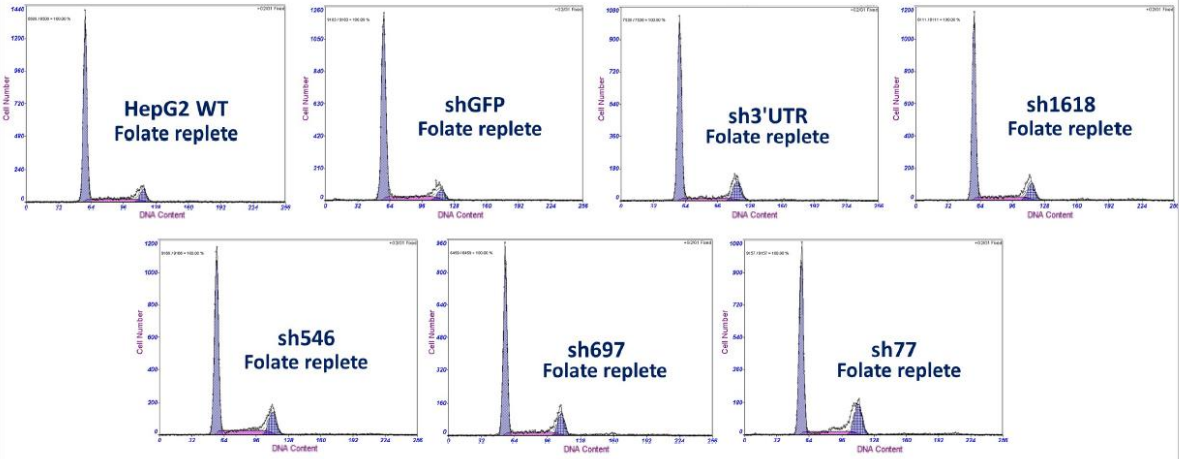

Supplemental Figure 3 cont. Original flow cytometry data for selected Table 1B (with raw data)

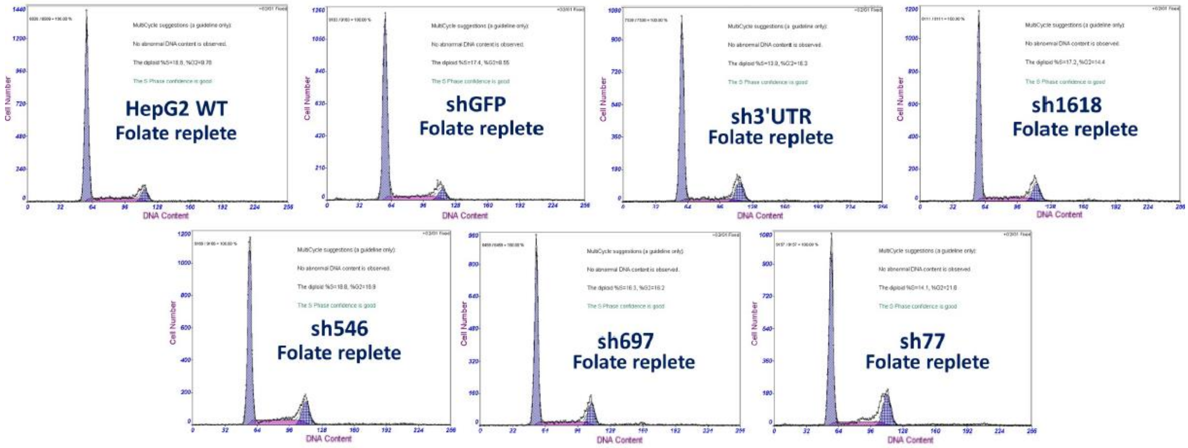

Supplemental Figure 4. Original flow cytometry data for selected Table 3A (with raw data)

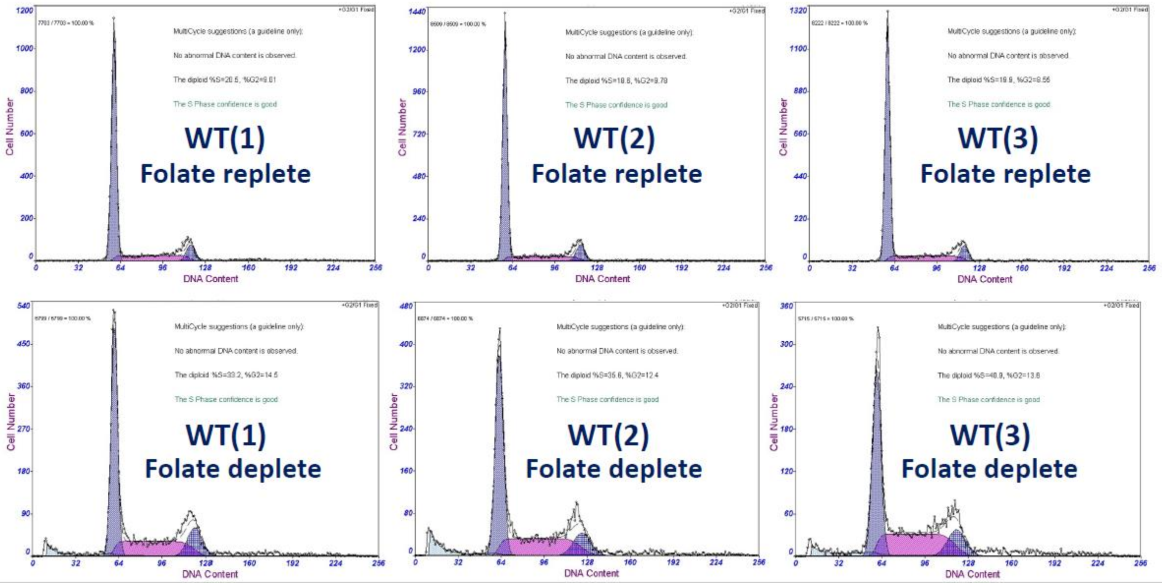

Supplemental Figure 4. Original flow cytometry data for selected Table 3A (with raw data)

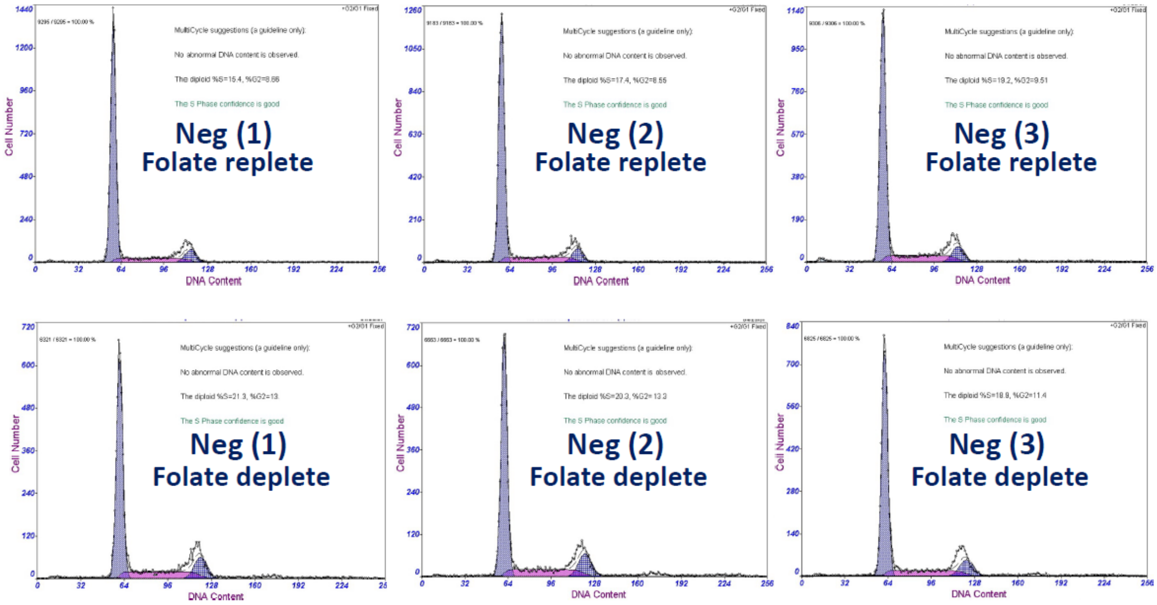

Supplemental Figure 4. Original flow cytometry data for selected Table 3A (with raw data)

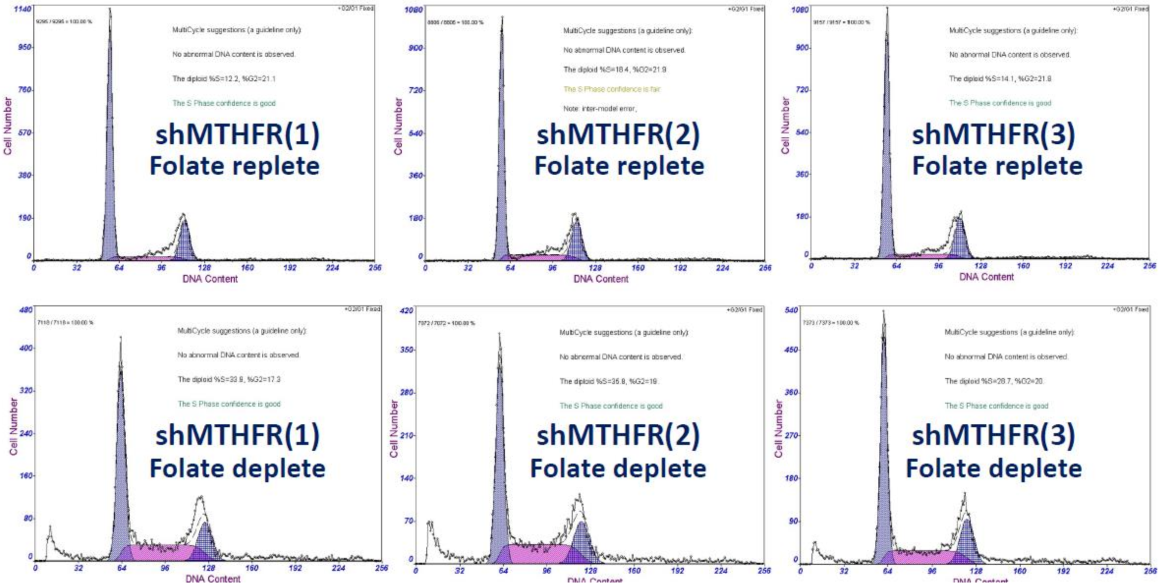

Supplement: Supplementary file 1 [file ijms-22-09392-s001.zip › ijms-1304950-supplementary.pdf]
